# Circ72688 Drives Breast Cancer Invasion and Metastasis via the miR-654-5p/ORAI2 Axis

**DOI:** 10.32604/or.2026.073081

**Published:** 2026-04-22

**Authors:** Haojie Yang, Zicong Tan, Zihao Liu, Kang Chen, Ning Liufu, Fengtao Ji

**Affiliations:** 1 Department of Anesthesia, Sun Yat-sen Memorial Hospital, Sun Yat-sen University, Guangzhou, China; 2 Guangdong Provincial Key Laboratory of Malignant Tumor Epigenetics and Gene Regulation, Guangdong-Hong Kong Joint Laboratory for RNA Medicine, Medical Research Center, Sun Yat-sen Memorial Hospital, Sun Yat-sen University, Guangzhou, China; 3 Department of Breast Surgery, The Third Affiliated Hospital of Kunming Medical University, Yunnan Cancer Hospital, Peking University Cancer Hospital Yunnan, Kunming, China

**Keywords:** Circular RNA, breast cancer, hsa-miR-654-5p, ORAI2, metastasis

## Abstract

**Background:**

Circular RNAs (circRNAs) play a crucial role in the progression of malignant tumors such as breast cancer.

**Methods:**

A circRNA microarray was used to detect key circRNAs in breast cancer. Expression of Circ72688 was verified by quantitative reverse transcription PCR (qRT-PCR) and fluorescence *in situ* hybridization (FISH) assay. Transwell assay and *in vivo* assay were conducted to prove the function of Circ72688. Exploring the downstream mechanism, dual-luciferase reporter assay, western blotting, and tissue microarray were performed.

**Results:**

We sequenced and identified a circRNA, hsa-circ-0072688, also known as Circ72688. Our research found that Circ72688 enhanced tumor metastasis both *in vivo* and *in vitro*. Mechanistically, Circ72688 acted as a molecular sponge for hsa-miR-654-5p, thereby affecting the transcription of ORAI2. The expression levels of Circ72688 and ORAI2 were significantly higher in breast cancer tissues compared to adjacent normal tissues, and a notable positive correlation could be seen between them.

**Conclusions:**

In summary, our study demonstrated the critical role of Circ72688 in breast cancer invasion and metastasis, and how Circ72688 partially regulated ORAI2 through hsa-miR-654-5p. Targeting Circ72688 might be a potential therapeutic strategy for breast cancer.

## 1 Introduction

Breast cancer maintains its status as the most prevalent malignancy among females globally, simultaneously topping the charts in terms of mortality rates [1,2]. Clinically, it accounts for roughly 24.5% of all newly diagnosed female cancer cases and contributes to 15.5% of cancer-related deaths in women, solidifying its position as the primary driver of cancer-related burden in this population [1]. Metastatic breast cancer remains predominantly incurable, and over 90% of breast cancer-associated deaths are directly linked to distant metastasis [3–5]. Though comprehensive treatment strategies have markedly improved long-term survival in recent years, the inherent high heterogeneity still leads to considerable variability in treatment responses across individual patients [6–10]. Therefore, identifying novel molecular targets is of great importance for the development of more effective therapeutic strategies.

Circular RNAs (circRNAs) are a class of endogenous non-coding RNAs characterized by a covalently closed-loop structure formed by joining the 3^′^ and 5^′^ ends of the RNA molecule [11–14]. This circular conformation confers resistance to degradation by endogenous and exogenous nucleases, allowing circRNAs to stably exist in various tumor tissues and bodily fluids, including breast cancer [15–17]. Numerous studies have demonstrated that cytoplasmic circRNAs regulate tumor cell proliferation, metastasis, and drug resistance mainly through three mechanisms: (i) acting as miRNA sponges to regulate the transcription of downstream target genes; (ii) interacting with proteins to influence their stability or the transcription of target genes; and (iii) serving as templates for translation to produce peptides that affect tumor progression [18–23].

Previous studies have shown that a disintegrin and metalloproteinase with thrombospondin motifs 6 (ADAMTS6) can transcribe multiple circRNAs. For instance, it generates hsa-circ-0072688 in esophageal squamous cell carcinoma (ESCC) [24], intrahepatic cholangiocarcinoma (ICC) [25], and glioblastoma (GBM) [26], as well as hsa-circ-0004418 and hsa-circ-0072676 in non-small-cell lung cancer (NSCLC) [27]. In addition, ADAMTS6 can produce hsa-circ-000866 under conditions of IL-1β-induced chondrocyte apoptosis and in osteoarthritic chondrocytes [28,29]. Although circRNAs derived from ADAMTS6 have been implicated in several malignancies, the biological function of hsa-circ-0072688 in breast cancer has not yet been explored.

This study is dedicated to uncovering the possibly significant circRNA for breast cancer therapy. In our preliminary RNA sequencing analysis of breast cancer tissues, hsa-circ-0072688 was identified as one of the most significantly upregulated circRNAs. Given that ADAMTS6-derived circRNAs have been implicated in tumor progression across multiple cancer types, we hypothesized that Circ72688 may also exert functional roles in breast cancer.

## 2 Methods

### 2.1 circRNA Microarray Detection

circRNA expression profiling was conducted by Aksomics Inc. (Shanghai, China). Total RNA was extracted from human mammary epithelial cells (HMEC) and breast cancer cell lines (MCF-7, SKBR3, and MDA-MB-231) using TRIzol reagent (15596026, Invitrogen, California, USA). RNA concentration and purity were assessed using a NanoDrop 2000 spectrophotometer (ND-2000, Thermo Fisher Scientific, Massachusetts, USA), and RNA integrity was evaluated using the Agilent 2100 Bioanalyzer (G2939BA, Agilent Technologies, California, USA). Samples with an RNA Integrity Number (RIN) ≥ 7.0 were selected for subsequent analysis. Ribosomal RNA (rRNA) was removed using the RiboMinus kit (K155003, Thermo Fisher Scientific, Massachusetts, USA), after which the remaining RNA was amplified and labeled with Cy3 fluorescent dye using the Aksomics Fluorescent Linear RNA Labeling Kit. The labeled cRNA was then hybridized onto the Human Circular RNA Array V2.0 (Aksomics). Following hybridization and washing, slides were scanned using the Agilent G2505C microarray scanner. Raw signal intensities were extracted using Agilent Feature Extraction software (version 10.7.1.1). Quantile normalization of the raw data was performed, and differential expression analysis was conducted using R software (version 4.1.3). circRNAs with a fold change ≥2.0 and *p* < 0.05 were considered significantly differentially expressed.

### 2.2 Cell Lines

Breast cancer cell lines MDA-MB-231, MCF-7, and SK-BR-3 were obtained from the American Type Culture Collection (ATCC, Manassas, VA, USA), and normal human mammary epithelial cells (HMECs) were purchased from Procell (Wuhan, China). All cell lines were cultured according to the supplier’s protocols. Lentiviral particles for Circ72688 overexpression were purchased from GenePharm (Shanghai, China), and transduction was performed using Polybrene (10 μg/mL) following the manufacturer’s instructions. Stable cell lines were established via selection with puromycin (2 μg/mL). For bioluminescence imaging, cells were transfected with luciferase-expressing vectors (Igebio, Guangzhou, China) and selected using neomycin (300 μg/mL).

### 2.3 Clinical Specimen

Tissue microarrays containing breast cancer tissues (Cat. No. Js W 03 01) and adjacent normal breast tissues (Cat. No. Js W 09 01) were purchased from Outdo Biotech (Shanghai, China), a certified commercial supplier of human clinical specimens. According to the provider’s documentation, all samples were obtained with written informed consent from donors, anonymized to remove personal identifiers, and collected under approval from the respective institutional ethics committee (Human Genetic Resource Administration of China, [2002] BC0020). All procedures complied with the Declaration of Helsinki and relevant national ethical guidelines. Two replicate microarrays of each type were used: one for *in situ* hybridization (ISH) to evaluate the expression level and spatial distribution of circRNA Circ72688, and the other for immunohistochemistry (IHC) to assess ORAI2 protein expression and localization. As the tissue microarrays were commercially acquired with ethical approval at the source institution, and are widely used in oncology research [30,31], additional approval from our institutional review board was not required.

### 2.4 Total RNA Extraction

After discarding the culture medium, cells were washed twice with ice-cold phosphate-buffered saline (PBS; C0221B, Beyotime, Shanghai, China). Following complete removal of PBS, TRIzol reagent (15596026, Thermo Fisher Scientific, Massachusetts, USA) was added, and the cells were resuspended by repeated pipetting. Samples were incubated at room temperature for 15 min to ensure complete lysis, followed by centrifugation at 12,000× *g* for 10 min at 4°C. The upper aqueous phase was carefully transferred to a new tube, mixed with chloroform (T_701ICN19400225, Thermo Fisher Scientific, Massachusetts, USA), incubated for 3 min at room temperature, and centrifuged at 12,000× *g* for 15 min at 4°C. The clear aqueous phase was then mixed with isopropanol (T036181000, Thermo Fisher Scientific, Massachusetts, USA), incubated at room temperature for 10 min, and centrifuged at 12,000× *g* for 10 min at 4°C to precipitate RNA. After discarding the supernatant, the pellet was washed with 75% ethanol at 4°C and centrifuged at 7500× *g* for 5 min. Finally, the ethanol was completely removed, and the RNA pellet was dissolved in 20 μL of nuclease-free water. RNA concentration and purity were measured using the NanoDrop One spectrophotometer (Thermo Fisher Scientific, Massachusetts, USA). Extracted RNA samples were stored at −80°C until further use.

### 2.5 RNase R Digestion Assay

A total of 2 μg of extracted RNA from breast cancer cells was equally divided into two aliquots (1 μg each). One aliquot served as the untreated control, while the other was treated with 0.05 μL RNase R enzyme and 0.7 μL Reaction Buffer (R0301, GeneSeed, Guangzhou, China). The final reaction volume for both the RNase R digestion and control groups was adjusted to 7 μL with nuclease-free water. The reaction mixtures were incubated at 37°C for 15 min in a PCR thermocycler. Following incubation, samples were immediately cooled on ice for subsequent analysis.

### 2.6 Actinomycin D Experiment

Breast cancer cells were seeded into six-well plates at a density of 1 × 10^6^ cells per well and incubated until they adhered to the plate surface. One well was designated as the untreated control group, while the remaining wells were assigned to the experimental groups. Actinomycin D (HY-17559, MedChemExpress, Shanghai, China) was added to the experimental wells at a final concentration of 2 μg/mL, whereas the control group received no treatment. Total RNA was extracted from the control well immediately at time 0 h, and from the actinomycin D–treated wells at 4, 8, 12, and 24 h, respectively.

### 2.7 RNA Cytoplasmic and Nuclear Fractionation

Cytoplasmic and nuclear RNA separation was performed using the PARIS^™^ Kit (AM1921, Thermo Fisher Scientific, Massachusetts, USA). After removing the culture medium, cells were washed twice with ice-cold PBS. Cell Disruption Buffer (100 µL per 1 × 10^6^ cells) was added, gently pipetted to resuspend, and incubated on ice for 5 min. An equal volume of Cell Fractionation Buffer was then added, mixed thoroughly, and centrifuged at 500× *g* for 5 min at 4°C. The supernatant (cytoplasmic fraction) and pellet (nuclear fraction) were collected separately. Cytoplasmic RNA was extracted by adding RNA Cell Disruption Buffer to the supernatant, while nuclear RNA was isolated by resuspending the pellet in RNA Cell Disruption Buffer. All procedures were performed according to the manufacturer’s protocol. RNA pellets were dissolved in 20 µL of nuclease-free water, and RNA concentration and purity were measured using a NanoDrop One spectrophotometer (Thermo Fisher Scientific, Massachusetts, USA). All RNA samples were stored at −80°C until further use. Total RNA was reverse transcribed into complementary DNA (cDNA) using the Evo M-MLV Reverse Transcription Premix (AG11706, AGbio, Changsha, China). qRT-PCR was performed using the Hieff qPCR SYBR Green Master Mix (11201ES08, Yeasen, Shanghai, China) on a real-time PCR system. Relative gene expression levels were calculated using the 2^−ΔΔCt^ method, as described in our previous study [32]. Primer sequences used in this study are listed below (Table 1):

**Table 1:** Primer sequences for Circ72688, mADAMTS6, ORAI2 and GAPDH.

	Forward Primer Sequence	Reverse Primer Sequences
Circ72688	5^′^-GGGATTGCCCACCACGATAA-3^′^;	5^′^-TCCAACATCCTGCACTTTCTTC-3^′^
mADAMTS6	5^′^-ATCACTCGAACTGGCAGTGG-3^′^;	5^′^-GTCTTTGGACACCTCCAGCA-3^′^
ORAI2	5^′^-TGGCGGAAGCTCTACCTGAG-3^′^;	5^′^-CGGGTACTGGTACTGCGTC-3^′^
GAPDH	5^′^-TGCACCACCAACTGCTTAGC-3^′^;	5^′^-TGCACCACCAACTGCTTAGC-3^′^

### 2.8 Western Blotting

Cells were harvested and lysed on ice using RIPA buffer (P0013B, Beyotime, Shanghai, China) supplemented with protease inhibitors (5871S, Cell Signaling Technology, Massachusetts, USA) for 30 min. Lysates were centrifuged at high speed, and the supernatants were collected for protein quantification. Equal amounts of protein were separated by SDS-PAGE (RFZ-RP-5025, Beyotime, Shanghai, China) and transferred onto PVDF membranes (IPVH00010, Merck, Darmstadt, Germany). After blocking, membranes were incubated overnight at 4°C with primary antibodies against ORAI2 (Abcam, ab155216;1:1000) diluted in blocking buffer. Following three washes with TBST, membranes were incubated with HRP-conjugated secondary antibodies (Cell Signaling Technology, Cat. 58802;1:5000) for 1 h at room temperature. Protein bands were detected using an ECL reagent (Beyotime) and visualized using a chemiluminescence imaging system (Syngene G: Box Chemi XT4, USA).

### 2.9 Transwell Migration and Invasion Assays

Transwell chambers with an 8-μm pore size (353097, BD Biosciences, USA) were used for migration and invasion assays. For the invasion assay, the upper surface of the membrane was coated with 50 μL Matrigel (354234, Corning, New York, USA) diluted in serum-free medium and allowed to polymerize overnight at 4°C. Uncoated chambers were used for migration assays. Transfected cells were resuspended in serum-free DMEM at a concentration of 1 × 10^6^ cells/mL, and 200 μL of the suspension was added to the upper chamber. The lower chamber was filled with 1 mL of DMEM containing 10% fetal bovine serum (FBS) to serve as a chemoattractant. After incubation at 37°C in 5% CO_2_ for 48 h (invasion assay) or 24 h (migration assay), non-migrated cells remaining on the upper surface of the membrane were gently removed using cotton swabs. Cells that migrated or invaded to the lower surface were fixed with 4% paraformaldehyde for 20 min, stained with 0.1% crystal violet for 15 min, and imaged under an Olympus IX83 microscope (Olympus, Tokyo, Japan). The number of migrated or invaded cells was quantified by counting cells in five randomly selected microscopic fields.

### 2.10 In Vivo Lung Metastasis Model

Female BALB/c nude mice (4–5 weeks old) were purchased from the Sun Yat-sen University Laboratory Animal Center (Guangzhou, China). A total of 20 mice were divided into 4 groups (MDA-MB-231 Vector, MDA-MB-231sh-Circ72688, MDA-MB-468 Vector, MDA-MB-468 sh-Circ72688). All mice were raised in a Specific Pathogen-Free (SPF) environment and in non-toxic, corrosion-resistant, and sterilized cages at 24°C–26°C. Mice were fed with sterilized full-price feed for SPF mice and sterile water. All animal procedures were approved by the Animal Ethics Committee of Sun Yat-sen Memorial Hospital of Sun Yat-sen University (Approval No. BAP20241003) and conducted in accordance with institutional guidelines and the ARRIVE standards. The lung metastasis model was established as previously described [21,22]. Briefly, 2 × 10^5^ cells were suspended in 200 μL of PBS and injected into the tail vein of nude mice. After 28 days, the mice were anesthetized and intraperitoneally injected with _D_-Luciferin (15 mg/mL) for bioluminescence imaging. Lung metastases were detected using an *in vivo* imaging system (IVIS 200, Massachusetts, USA) to quantify bioluminescent signal intensity. Following imaging, lung tissues were excised, fixed in 4% paraformaldehyde, and embedded in paraffin for subsequent histological and immunohistochemical analyses.

### 2.11 Hematoxylin and Eosin (HE) Staining

Mouse lung tissues were fixed in 10% neutral-buffered formalin for 48 h, dehydrated in graded ethanol, cleared in xylene, and embedded in paraffin. Tissue sections (4 μm thick) were cut and baked at 60°C for 1 h. After deparaffinization in xylene and rehydration through graded ethanol solutions, sections were rinsed in distilled water, stained with hematoxylin for 5 min, washed under running tap water for 1 min, counterstained with eosin for 2 min, and subsequently dehydrated. Slides were then cleared in xylene and mounted with neutral resin. Histological images were captured using a light microscope (IX83; Olympus, Tokyo, Japan). Tissue sampling and sectioning procedures followed standard protocols for HE staining.

### 2.12 Immunohistochemistry (IHC)

Tissue microarray sections were deparaffinized, rehydrated, and subjected to antigen retrieval in 10 mM citrate buffer (pH 6.0), followed by washing in PBS. Endogenous peroxidase activity was blocked using 5% normal goat serum for 30 min at room temperature. The sections were then incubated with anti-ORAI2 primary antibody (AB155216, Abcam, Cambridge, UK; 1:200) overnight at 4°C. After PBS washing, the sections were incubated with HRP-conjugated secondary antibody (1:500) for 1 h at room temperature. Color development was performed using DAB substrate for 2 min, and nuclei were counterstained with hematoxylin for 1 min. Finally, sections were washed, dehydrated, cleared in xylene, and mounted. IHC staining was performed to assess ORAI2 protein expression and spatial distribution in breast cancer tissues and adjacent normal epithelial tissues. Staining was evaluated based on both staining intensity and the percentage of positively stained tumor cells in the cytoplasmic or nuclear compartments.

Staining intensity was scored as: 0 = negative, 1 = weak (1+), 2 = moderate (2+), and 3 = strong (3+). The percentage of positive tumor cells was scored as: 0 (<1%), 1 (1%–25%), 2 (26%–50%), 3 (51%–75%), and 4 (76%–100%). An IHC score for each specimen was calculated by multiplying the intensity score by the positivity score, resulting in a total score ranging from 0% to 300%. For survival analysis, ORAI2 expression was categorized as high (>90%) or low (≤90%) based on the total score. Scoring criteria and additional procedures were performed as previously described in our published studies [33,34].

### 2.13 In Situ Hybridization (ISH) on Tissue Microarrays

Paraffin-embedded tissue microarrays were baked at 65°C for 1 h, deparaffinized in xylene, and rehydrated through graded ethanol. Antigen retrieval was performed according to standard protocols, followed by enzymatic digestion with Proteinase K (20 μg/mL; Thermo Fisher Scientific, Massachusetts, USA) for 15 min at room temperature to enhance probe penetration. The DIG-labeled Circ72688-specific probe (Qiagen, Germany) was pretreated according to the manufacturer’s instructions and hybridized to the tissue sections. After hybridization, slides were washed twice with 2× SSC/50% formamide and twice with 1× SSC (ST463, Beyotime, Shanghai, China). Endogenous peroxidase activity was quenched using 3% H_2_O_2_ in PBS for 10 min, followed by blocking with 5% normal goat serum for 30 min at room temperature. Sections were then incubated with anti-DIG antibody (11093274910, Roche, Basel, Switzerland; 1:200) for 1 h at room temperature. After PBS washing, color development was performed using NBT/BCIP substrate until a visible signal appeared. Finally, sections were washed, dehydrated, cleared, and mounted. ISH was conducted to determine the expression level and spatial distribution of Circ72688 in breast cancer and adjacent normal tissues. Staining results were evaluated using a semi-quantitative scoring system based on both staining intensity (0, 1+, 2+, 3+) and the percentage of positively stained tumor cells (0: <1%; 1: 1%–25%; 2: 26%–50%; 3: 51%–75%; 4: 76%–100%). The final ISH score for each specimen was calculated by multiplying the intensity score by the positivity score, yielding a score range of 0%–300%. For survival analysis, cases with cytoplasmic or nuclear staining scores >10% were classified as high expression, while those with scores ≤10% were designated as low expression. Probe sequence (5^′^ → 3^′^): 5DiGN/TGGCCATTTTTTAACCTAGTAA/3DiG-N/ (Qiagen, Cat. No. 339115YCD0076297-BCG).

### 2.14 Fluorescence In Situ Hybridization (FISH)

Breast cancer cells were fixed with 4% paraformaldehyde for 15 min, permeabilized with 0.5% Triton X-100 for 10 min, and pre-hybridized in hybridization buffer (2× SSC, 50% formamide, 10% dextran sulfate, and 1× Denhardt’s solution) at 37°C for 30 min. A Cy3-labeled Circ72688-specific probe (GenePharma, Shanghai, China) was diluted to 1 μM in hybridization buffer and applied to the samples, followed by overnight hybridization at 37°C in a humidified chamber. After hybridization, slides were washed twice in 2× SSC containing 50% formamide at 42°C for 10 min and subsequently washed twice in 1× SSC at room temperature for 5 min each. Nuclei were counterstained with DAPI, and coverslips were mounted using an antifade reagent. Fluorescent signals were captured using a confocal microscope (FV3000; Olympus, Tokyo, Japan) under identical acquisition settings across all samples. FISH was used to determine the expression level and subcellular localization of Circ72688 in breast cancer cells. All procedures were performed according to the GenePharma RNA FISH kit (GenePharma, Shanghai, China). Probe sequences are provided in Table 2.

**Table 2:** Primer sequences for Circ72688 in FISH.

Circ72688	5^′^-GCCATTTTTTAACCTAGTAATAAGAACTGC-3^′^
	5^′^-CATTTTTTAACCTAGTAATAAGAACTGCAT-3^′^
	5^′^-TTTAACCTAGTAATAAGAACTGCATTAT-3^′^

### 2.15 Data Acquisition and Preprocessing of TCGA-BRCA miRNA-Seq and RNA-Seq Expression Profiles

Raw miRNA isoform read counts and normalized reads per million (RPM) values from miRNA-Seq, as well as raw gene read counts and normalized fragments per kilobase per million (FPKM) values from RNA-Seq, were downloaded from the Genomic Data Commons (GDC) portal (https://portal.gdc.cancer.gov/) for the TCGA-BRCA cohort. The miRNA-Seq dataset consisted of 1189 samples (1085 tumor and 104 normal), and the RNA-Seq dataset included 1211 samples (1098 tumor and 113 normal). Low-abundance features (miRNAs or genes with fewer than 10 read counts in more than 50% of the samples) were excluded prior to downstream analysis. Expression raw counts were normalized and processed for differential expression analysis using the DESeq2 package (version 1.30.0) in R (version 4.0.3). Size factor normalization was performed using the median-of-ratios method, and the Wald test was applied for statistical significance testing. Differentially expressed miRNAs and genes between tumor and normal samples were defined by |log_2_ fold change| > 0.586 (corresponding to approximately 1.5-fold change) and *p* < 0.05.

### 2.16 Prediction of miRNAs Bound to Cir72688 and Their mRNA Targets

The genomic coordinates of Circ72688 were obtained from circBase (accession ID: hsa-circ-0072688) and used to retrieve its full-length sequence from the hg19 human reference genome using the UCSC Table Browser. Potential miRNA binding sites on Circ72688 were predicted using two independent bioinformatic tools: RNAhybrid (v2.1.2) with default seed constraints and a minimum free energy (MFE) threshold of ≤ −20 kcal/mol, and miRanda (v3.3a) with a score threshold ≥ 140 and binding energy ≤ −20 kcal/mol. Only miRNAs predicted by both tools were retained. To further narrow down the candidates to biologically relevant miRNAs whose expression trends were inversely correlated with Circ72688, we intersected this set with miRNAs significantly downregulated in TCGA-BRCA tumor tissues (|log_2_ fold change| > 0.586, *p* < 0.05), resulting in 32 candidate miRNAs. Next, potential mRNA targets of these 32 miRNAs were predicted using three complementary databases: TargetScan, miRDB, and miRTarBase. Only genes present in all three databases were considered high-confidence miRNA targets. These predicted targets were then intersected with genes significantly upregulated in TCGA-BRCA tumor tissues (|log_2_ fold change| > 0.586, *p* < 0.05). MiRNAs with no matched upregulated targets were excluded, ultimately yielding 22 miRNAs paired with 115 mRNA targets.

### 2.17 Construction of the Least Absolute Shrinkage and Selection Operator (LASSO) Regression Model

Normalized expression matrices of the 22 candidate miRNAs and 115 mRNAs were extracted from the TCGA-BRCA cohort. Expression values were log_2_-transformed (where necessary) and standardized to z-scores before model construction. A LASSO-penalized Cox proportional hazards regression model was implemented using the *glmnet* package (v4.1-1) in R (v4.0.3). Ten-fold cross-validation (*cv.glmnet*) was employed to determine the optimal regularization parameter (λ) that minimized the mean cross-validated partial likelihood deviance. At the λ corresponding to the minimum deviance, seven miRNAs and eight mRNAs retained non-zero coefficients, namely: miRNAs: hsa-miR-511-5p, hsa-miR-519d-3p, hsa-miR-370-3p, hsa-miR-5683, hsa-miR-433-3p, hsa-miR-654-5p, hsa-miR-520g-3p. mRNAs: BAMBI, CERCAM, KCTD15, KRAS, MORF4L2, ORAI2, PLS1, RACGAP1. A patient-specific risk score was calculated as a linear combination of the expression levels of these 15 features weighted by their corresponding regression coefficients: Risk score = β_1_X_1_ + β_2_X_2_ + … + β_n_X_n_, where *β* represents the regression coefficient, and *X* denotes the expression level of the corresponding gene or miRNA.

### 2.18 Time-Dependent Receiver Operating Characteristic (ROC) Curve Analysis

Time-dependent ROC curves at 1, 3, and 5 years were generated using the *multiROC* package (v1.1.0) in R (v4.0.3). The risk scores calculated for all 1098 TCGA-BRCA tumor samples were used as continuous predictors. For each time point, sensitivity and specificity were computed across the full range of risk score values, and the corresponding ROC curves were plotted. The area under the curve (AUC) was calculated to assess the predictive performance at each interval. The risk score value yielding the highest Youden index was selected as the optimal cutoff and subsequently applied to stratify patients into high-risk and low-risk groups.

### 2.19 Survival Analysis

A total of 1098 TCGA-BRCA tumor samples and tissue microarray data were included in Kaplan–Meier (KM) survival analysis. KM survival curves were generated using the survminer package in R, and differences between high-risk and low-risk groups were assessed using the two-sided log-rank test. Univariate Cox proportional hazards regression was performed to estimate hazard ratios (HRs) and 95% confidence intervals (CIs) for the risk-score-based stratification. Additionally, each of the 15 LASSO-selected features (seven miRNAs and eight mRNAs) was individually dichotomized within the tumor cohort using two grouping strategies: (1) optimal cutoff identified by surv_cutpoint, and (2) median expression level. KM survival curves and log-rank tests were generated for each feature based on both grouping criteria. To visualize differential expression between tumor (n = 1098) and normal (n = 113) samples, boxplots for all 15 features were generated using *ggplot2*. Statistical differences in expression were assessed using the two-sided Wilcoxon rank-sum test, with *p* < 0.05 considered statistically significant.

### 2.20 Immune Infiltration Analysis by CIBERSORT

To evaluate differences in the tumor immune microenvironment between risk groups, immune cell composition was estimated using the CIBERSORT algorithm with the LM22 signature matrix (https://cibersort.stanford.edu/). Normalized FPKM expression values of all 1098 TCGA-BRCA tumor samples were used as input. CIBERSORT was run with 100 permutations in “absolute” mode to quantify the relative proportions of 22 immune cell subtypes per sample. Samples were stratified into high- and low-risk groups based on the optimal cutoff determined by the maximum AUC in the time-dependent ROC analysis. The infiltration levels of each of the 22 immune cell types were compared between the two risk groups using the two-sided Wilcoxon rank-sum test (wilcox.test in R), with *p* < 0.05 considered statistically significant. Significant differences were visualized using boxplots generated with *ggplot2* (v3.3.5), highlighting immune cell populations enriched in each risk group. This analytical approach was consistent with the method employed in our previous study.

### 2.21 Drug Sensitivity Prediction Using oncoPredict

To assess potential differences in drug sensitivity between risk groups, the oncoPredict R package was used to estimate the half-maximal inhibitory concentration (IC50) of 198 chemotherapeutic agents based on the Genomics of Drug Sensitivity in Cancer (GDSC) training dataset (https://www.cancerrxgene.org/). The gene expression matrix of all TCGA-BRCA tumor samples (n = 1098) served as the input for prediction. Drug sensitivity was inferred using ridge regression models trained on GDSC cell line data, following the default workflow in oncoPredict. After calculation of predicted IC50 values across all samples, patients were classified into high- and low-risk groups according to the optimal risk score cutoff determined by the time-dependent ROC analysis. For each drug, differences in predicted IC50 between the two risk groups were evaluated using the two-sided Wilcoxon rank-sum test. Boxplots were generated using ggplot2 to visualize compounds with differential sensitivity. Drugs with statistically significant IC50 differences (*p* < 0.05) were considered to demonstrate differential sensitivity associated with the risk signature.

### 2.22 Gene Set Variation Analysis (GSVA)

To investigate pathway-level functional differences between risk groups, Gene Set Variation Analysis (GSVA) was performed using the GSVA package (v1.44.5) in R. Three gene set collections were retrieved from the MSigDB database: Hallmark pathways, Gene Ontology (GO) terms, and Kyoto Encyclopedia of Genes and Genomes (KEGG) pathways. GSVA enrichment scores were calculated for each TCGA-BRCA tumor sample (n = 1098) using normalized FPKM expression data. Based on the optimal risk score cutoff determined by the time-dependent ROC analysis, tumor samples were classified into high- and low-risk groups. Groupwise comparisons of GSVA enrichment scores were conducted using the two-sided Wilcoxon rank-sum test (wilcox.test in R), with *p* < 0.05 considered statistically significant. The top differentially enriched GO terms and signaling pathways were visualized using pheatmap (v1.0.12) in the form of GSVA heatmaps.

### 2.23 Statistical Analysis

All experiments were performed independently with at least three biological replicates, and data were presented as the mean ± standard deviation (SD).. Survival analyses were conducted using the Kaplan–Meier method, and differences between groups were assessed using the log-rank test. For comparisons of continuous variables with normal distributions between two groups, two-tailed Student’s t-tests were applied. For non-normally distributed data, the Wilcoxon rank-sum test was used. A *p*-value of < 0.05 was considered statistically significant. The bioinformatics tools and statistical approaches employed in this study were consistent with those described in our previous publication [35].

## 3 Results

### 3.1 Identification and Validation of Circ72688 as a Highly Expressed circRNA in Breast Cancer

**Figure 1:**
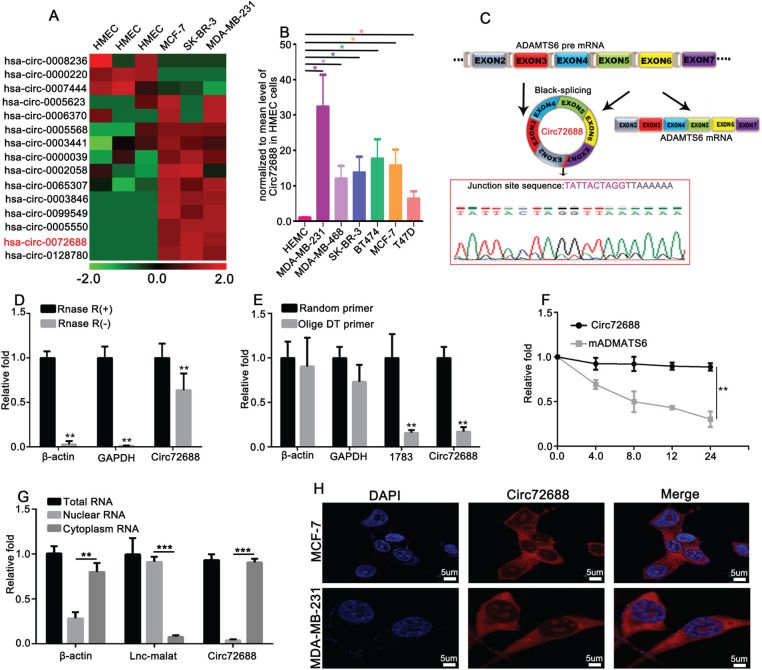
Screening of candidate circRNAs in breast cancer cell lines and characterization of Circ72688 (**A**) Heatmap showing the top differentially expressed circRNAs identified by circRNA microarray analysis in breast cancer cell lines and HMEC cells. (**B**) Relative expression levels of Circ72688 in various breast cancer cell lines determined by qRT-PCR. (**C**) Sanger sequencing confirming the back-splicing junction site of Circ72688 in MDA-MB-231 cells. (**D**) qRT-PCR analysis of β-actin, GAPDH, and Circ72688 before and after RNase R digestion, demonstrating the resistance of Circ72688 to RNase R degradation. (**E**) qRT-PCR analysis of β-actin, GAPDH, circRNA001783, and Circ72688 using Random primers and Oligo(DT) primers, confirming the circular structure of Circ72688. (**F**) Stability analysis of Circ72688 after actinomycin D treatment, showing higher stability compared with linear mRNA. (**G**) Subcellular localization of Circ72688 in the cytoplasmic and nuclear fractions using the PARIS kit and qRT-PCR. (**H**) RNA FISH analysis showing cytoplasmic localization of Circ72688 in MCF-7 and MDA-MB-231 cells. Images were captured using a confocal laser scanning microscope equipped with a 60× oil-immersion objective. *Statistical significance: *p < 0.05, **p < 0.01, ***p < 0.001. qRT-PCR: Quantitative Reverse Transcription Polymerase Chain Reaction; FISH: Fluorescence *In Situ* Hybridization; DT: Deoxythymidine; GAPDH: Glyceraldehyde-3-Phosphate Dehydrogenase.

Using human mammary epithelial cells (HMEC) and breast cancer cell lines (MCF-7, SKBR3, and MDA-MB-231) for circRNA microarray profiling, we generated a heatmap of the top 15 differentially expressed circRNAs (Fig. 1A). Validation of the sequencing results by qRT-PCR confirmed that Circ72688 exhibited the most significant upregulation among the candidates and was therefore selected for further investigation (Fig. 1B). According to circBASE, hsa-circ-0072688 (Circ72688) is derived from the back-splicing between exons 2 and 7 of the ADAMTS6 pre-mRNA, resulting in a circular transcript of 1352 nucleotides. Sanger sequencing further confirmed the head-to-tail splicing junction of Circ72688 (Fig. 1C). To verify the circular structure of Circ72688, RNase R digestion assays were performed, demonstrating that Circ72688 was resistant to RNase R–mediated degradation, whereas linear ADAMTS6 mRNA (mADAMTS6) was significantly degraded (Fig. 1D). Additionally, qRT-PCR using both Random and Oligo(dT) primers, with circ001783 as a positive control [36], confirmed the absence of a poly(A) tail and the circular nature of Circ72688 (Fig. 1E). Actinomycin D treatment further demonstrated that Circ72688 exhibited greater stability than mADAMTS6 (Fig. 1F). To assess the subcellular localization of Circ72688, we performed qRT-PCR using β-actin and lncRNA-MALAT1 as cytoplasmic and nuclear controls, respectively [37,38]. The results showed that Circ72688 was predominantly localized in the cytoplasm (Fig. 1G). This finding was further confirmed by RNA FISH assays, which revealed strong cytoplasmic fluorescence signals for Circ72688 (Fig. 1H). Collectively, these findings confirm that Circ72688 is a bona fide circular RNA that is highly expressed and predominantly localized in the cytoplasm of breast cancer cells.

### 3.2 Circ72688 Promotes Migration and Invasion of Breast Cancer Cells In Vitro and In Vivo

**Figure 2:**
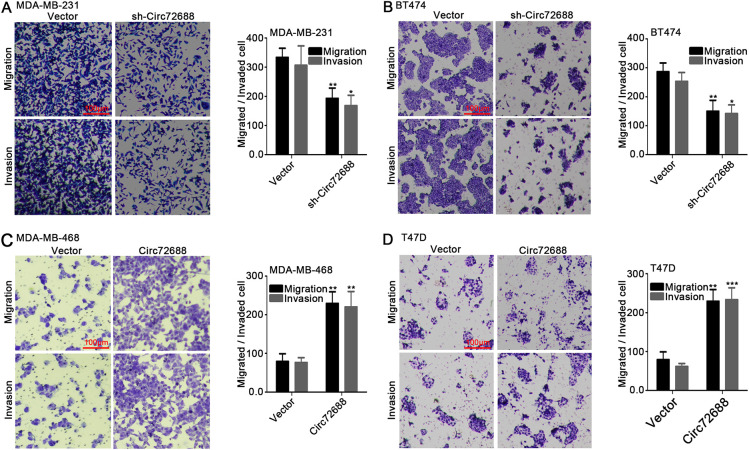

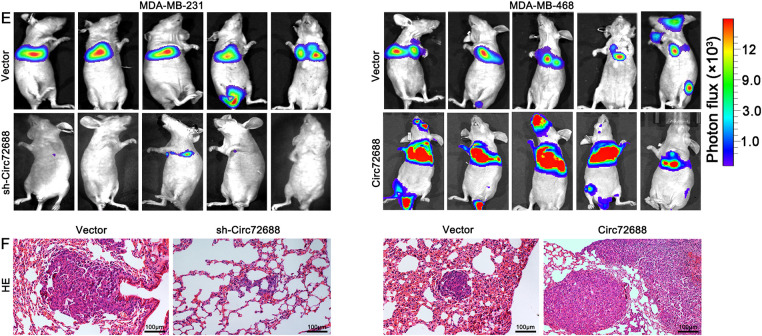
Effect of Circ72688 on the migratory and invasive abilities of breast cancer cells *in vitro* and tumor metastasis *in vivo*. (**A**) Representative Transwell images showing the migration and invasion of MDA-MB-231 cells following Circ72688 knockdown. (Scale bar: 100 μm) (**B**) Representative images of migration and invasion assays in BT474 cells with Circ72688 knockdown. (Scale bar: 100 μm) (**C**) Representative Transwell images showing enhanced migration and invasion of MDA-MB-468 cells following Circ72688 overexpression. (Scale bar: 100 μm) (**D**) Representative images of migration and invasion assays in T47D cells overexpressing Circ72688. (Scale bar: 100 μm) (**E**) Representative *in vivo* bioluminescence images of lung metastasis in nude mice injected with MDA-MB-231 (sh-Circ72688) or MDA-MB-468 (Circ72688-overexpression) cells. (**F**) Representative HE-stained histological images of lung metastatic lesions. Images were acquired under 10× magnification using a light microscope. *Statistical significance: *p < 0.05, **p < 0.01, ***p < 0.001. HE: Hematoxylin-Eosin.

To investigate the biological role of Circ72688 in breast cancer progression, Circ72688 was silenced in MDA-MB-231 (Fig. S1A) and BT474 (Fig. S1B) cells, while overexpression was established in MDA-MB-468 (Fig. S1C) and T47D (Fig. S1D) cells. Transwell assays revealed that knockdown of Circ72688 significantly inhibited the migratory and invasive abilities of MDA-MB-231 and BT474 cells (Fig. 2A,B). Conversely, overexpression of Circ72688 markedly enhanced migration and invasion in MDA-MB-468 and T47D cells (Fig. 2C,D). To further validate these findings *in vivo*, a lung metastasis model was established using MDA-MB-231 cells transfected with Circ72688 knockdown and MDA-MB-468 cells overexpressing Circ72688. After 35 days, bioluminescence imaging demonstrated a significantly weaker fluorescence signal in the sh-Circ72688 group compared with the corresponding vector control, whereas the Circ72688-overexpression group exhibited a markedly stronger signal than its control (Figs. 2E and S1E). Subsequent histological examination of lung tissues using HE staining showed fewer and smaller metastatic nodules in the sh-Circ72688 group, whereas the Circ72688-overexpression group displayed more extensive and larger metastatic lesions compared to the control groups (Fig. 2F). Collectively, these findings indicate that Circ72688 plays a pivotal role in promoting the migration and invasion of breast cancer cells both *in vitro* and *in vivo*.

### 3.3 Circ72688 Promotes Breast Cancer Progression by Sponging hsa-miR-654-5p to Partially Regulate ORAI2 Expression

**Figure 3:**
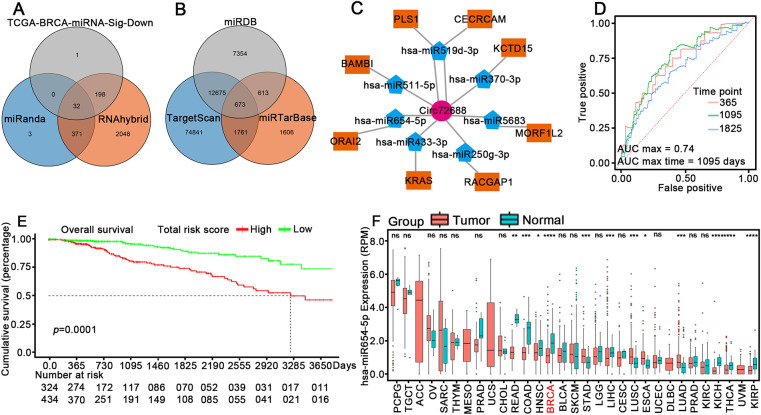

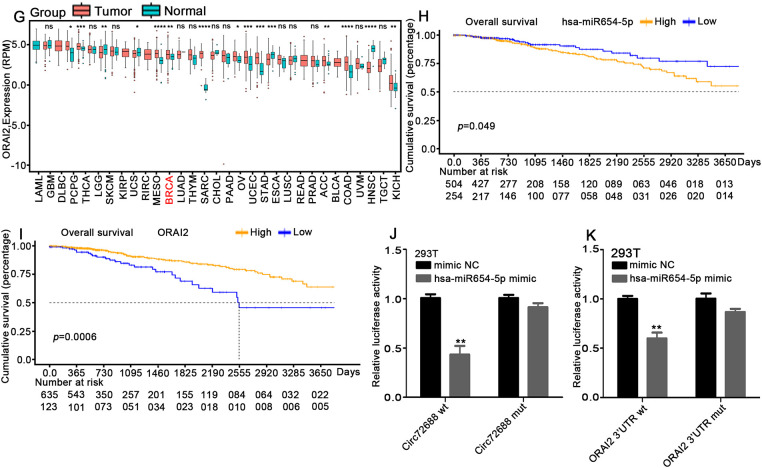
Circ72688 functions as a sponge for hsa-miR-654-5p to regulate ORAI2. (**A**) Venn diagram showing the intersection of miRNAs that are significantly downregulated in TCGA-BRCA tumor tissues and those predicted to bind Circ72688 by both miRanda and RNAhybrid. (**B**) Venn diagram showing the intersection of target mRNAs predicted by TargetScan, miRTarBase, and miRDB and significantly upregulated genes in TCGA-BRCA tumor samples. (**C**) Construction of the Circ72688–miRNA–mRNA ceRNA regulatory network. (**D**) Time-dependent ROC curve of the prognostic risk model constructed from Circ72688-associated miRNAs and mRNAs. (**E**) Kaplan–Meier curves showing overall survival of high- and low-risk groups based on risk scores in TCGA-BRCA. (**F**) Pan-cancer expression analysis of hsa-miR-654-5p. ns: not significant. *Statistical significance: *p < 0.05, **p < 0.01, ***p < 0.001, ****p < 0.0001 (**G**) Pan-cancer expression analysis of ORAI2. ns: not significant. *Statistical significance: *p < 0.05, **p < 0.01, ***p < 0.001, ****p < 0.0001 (**H**) Prognostic significance of hsa-miR-654-5p in breast cancer samples. (**I**) Prognostic significance of ORAI2 in breast cancer samples. (**J**) Dual-luciferase assay showing binding of hsa-miR-654-5p to Circ72688. *Statistical significance: **p < 0.01 (**K**) Dual-luciferase assay showing binding of hsa-miR-654-5p to the 3^′^UTR of ORAI2. *Statistical significance: **p < 0.01. wt: wild type; mut: mutation.

Previous studies have shown that cytoplasmic circRNAs primarily regulate gene expression through miRNA sponging, thereby modulating tumor cell behavior [17]. To further elucidate the regulatory mechanism of Circ72688, we first extracted its full sequence based on genomic coordinates (hg19) and predicted potential binding miRNAs using both miRanda and RNAhybrid algorithms. The intersection of these two prediction tools yielded a candidate set of miRNAs, which was further intersected with miRNAs significantly downregulated in TCGA-BRCA. This resulted in 32 candidate miRNAs showing an inverse expression trend with Circ72688, suggestive of potential sponge interactions (Fig. 3A). Next, mRNA targets of the 32 miRNAs were predicted using TargetScan, miRTarBase, and miRDB. Common targets across all three databases were intersected with significantly upregulated genes in TCGA-BRCA, yielding 115 mRNAs paired with 22 miRNAs (Fig. 3B). These miRNA–mRNA pairs formed a preliminary Circ72688-associated ceRNA network. To refine this regulatory network, expression profiles of the 22 miRNAs and 115 mRNAs were extracted from TCGA-BRCA samples and subjected to LASSO Cox regression modeling. Seven miRNAs and eight mRNAs were retained as core regulators associated with Circ72688 expression and prognosis (Fig. 3C). Based on these 15 ceRNA-related genes, a prognostic risk score model was constructed. Time-dependent ROC analysis demonstrated favorable predictive performance, with an AUC of 0.74 for 3-year overall survival (Fig. 3D). Kaplan–Meier survival analysis showed that the high-risk group had significantly poorer survival than the low-risk group (*p* < 0.001, Fig. 3E). In addition, individual factors within the model also showed significant prognostic relevance when grouped by median or optimal cutoff. Box plots further confirmed that these core regulatory genes were differentially expressed between tumor and normal tissues in TCGA-BRCA. We analyzed the differential expression of several miRNAs across multiple tumor types and their prognostic significance in breast cancer, including hsa-miR-370-3p (Fig. S2A,B), hsa-miR-433-3p (Fig. S2C,D), hsa-miR-520g-3p (Fig. S2E,F), hsa-miR-511-5p (Fig. S2G,H), hsa-miR-5683 (Fig. S2I,J), and hsa-miR-519d-3p (Fig. S2K,L). Similarly, we assessed the differential expression of mRNAs in various tumors and their prognostic significance in breast cancer, including MORF4L2 (Fig. S3A,B), CERCAM (Fig. S3C,D), PLS1 (Fig. S3E,F), KCTD15 (Fig. S3G,H), RACGAP1 (Fig. S3I,J), KRAS (Fig. S3K,L), and BAMBI (Fig. S3M,N). Further pan-cancer expression analysis revealed that hsa-miR-654-5p was significantly overexpressed in BRCA and multiple other malignancies (Fig. 3F), whereas ORAI2 was consistently downregulated across several cancer types (Fig. 3G). Both hsa-miR-654-5p and ORAI2 were significantly associated with breast cancer prognosis (Fig. 3H,I). Dual-luciferase reporter assays confirmed that hsa-miR-654-5p binds directly to Circ72688 (Fig. 3J) and to the 3^′^UTR of ORAI2 (Fig. 3K). To verify whether Circ72688 promotes breast cancer migration and invasion via hsa-miR-654-5p, rescue experiments were performed. Co-transfection of an hsa-miR-654-5p inhibitor partially reversed the inhibitory effects of Circ72688 knockdown on migration and invasion in MDA-MB-231 and BT474 cells (Fig. S4A,B). Conversely, co-transfection of an hsa-miR-654-5p mimic partially abrogated the promotive effects of Circ72688 overexpression in MDA-MB-468 and T47D cells (Fig. S4C,D). Western blot results further demonstrated that Circ72688 knockdown reduced ORAI2 protein expression in MDA-MB-231 and BT474 cells (Fig. S4E,F), whereas Circ72688 overexpression markedly increased ORAI2 expression in MDA-MB-468 and T47D cells (Fig. S4G,H). Importantly, the hsa-miR-654-5p inhibitor attenuated the downregulation of ORAI2 caused by Circ72688 knockdown (Fig. S4I,J), and the hsa-miR-654-5p mimic partially reversed the upregulation of ORAI2 induced by Circ72688 overexpression (Fig. S4K,L). Collectively, these findings indicate that Circ72688 promotes breast cancer cell migration and invasion by sponging hsa-miR-654-5p to regulate ORAI2 expression.

### 3.4 Circ72688 and ORAI2 are Significantly Upregulated in Breast Cancer Tissues

**Figure 4:**
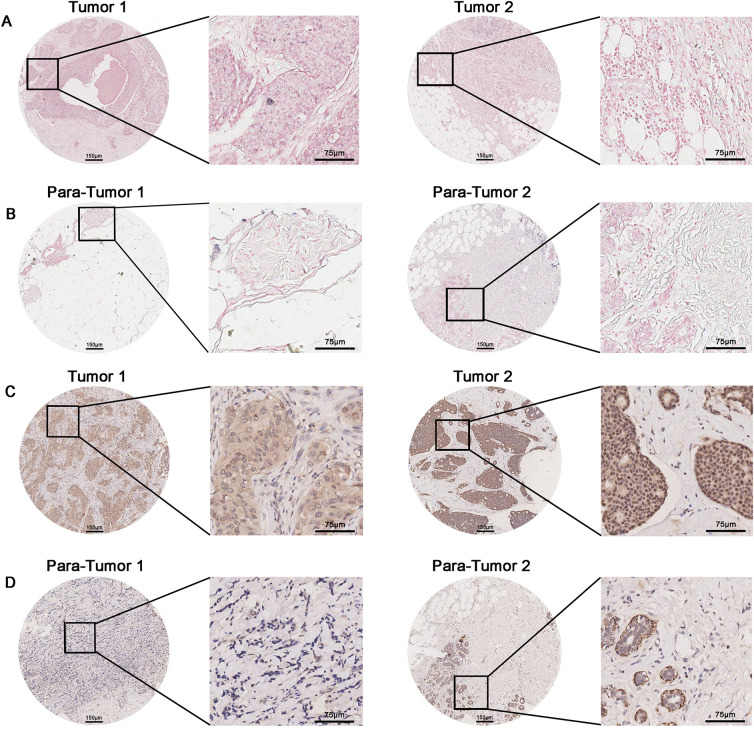

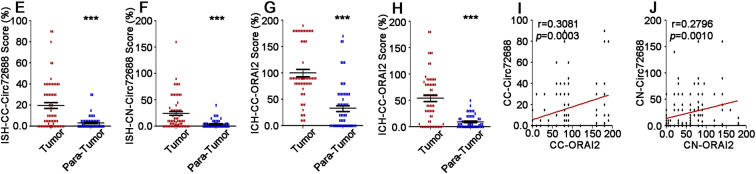
Circ72688 and ORAI2 are upregulated in breast cancer tissues and positively correlated with each other. (**A**) ISH detection of Circ72688 expression and subcellular localization in breast cancer tissues. (Scale bar: 150 μm) (**B**) ISH detection of Circ72688 expression and subcellular localization in adjacent normal breast tissues. (Scale bar: 150 μm) (**C**) IHC detection of ORAI2 expression and subcellular localization in breast cancer tissues. (Scale bar: 150 μm) (**D**) IHC detection of ORAI2 expression and subcellular localization in adjacent normal breast tissues. (Scale bar: 150 μm) (**E**) Cytoplasmic Circ72688 expression levels in breast cancer tissues analyzed by ISH. *Statistical significance: .***p < 0.001 (**F**) Nuclear Circ72688 expression levels in breast cancer tissues analyzed by ISH. *Statistical significance: ***p < 0.001 (**G**) Cytoplasmic ORAI2 expression levels in breast cancer tissues analyzed by IHC. 6 *Statistical significance: ***p < 0.001 (**H**) Nuclear ORAI2 expression levels in breast cancer tissues analyzed by IHC. *Statistical significance: ***p < 0.001 (**I**) Spearman correlation analysis showing the positive association between Circ72688 and ORAI2 expression in the cytoplasm. (**J**) Spearman correlation analysis showing the positive association between Circ72688 and ORAI2 expression in the nucleus. Histological images were captured using a light microscope (IX83; Olympus, Tokyo, Japan). Circular low-magnification ISH/IHC images were acquired at 2×, and square high-magnification images were captured at 20×. ISH: *In Situ* Hybridization; ICH: Immunohistochemistry.

To further explore the clinical relevance of Circ72688 and ORAI2, we examined their expression levels and spatial distribution in breast cancer and adjacent normal tissues using tissue microarrays. A total of 64 breast cancer samples were stratified into high- and low-expression groups based on Circ72688 and ORAI2 staining levels. ISH analysis revealed that Circ72688 expression in both the cytoplasm and nucleus was significantly higher in breast cancer tissues compared with adjacent normal tissues (Fig. 4A,B,E,F; Tables S1 and S2). However, Cytoplasmic and nuclear Circ72688 expression showed no significant correlation with clinicopathological parameters (Tables S3 and S4) or overall survival (OS) and disease-free survival (DFS) (Fig. S5A–D). Cox multivariate analysis further confirmed that neither cytoplasmic nor nuclear Circ72688 expression served as an independent prognostic factor, whereas age, pathological grade, and N stage did (Tables S5 and S6). IHC analysis demonstrated that ORAI2 expression in both the cytoplasm and nucleus was markedly higher in breast cancer tissues than in adjacent tissues (Fig. 4C,D,G,H; Tables S7 and S8). Although ORAI2 expression did not correlate significantly with clinicopathological parameters (Tables S9 and S10), high cytoplasmic ORAI2 expression was significantly associated with poorer OS and DFS (Fig. S5E,F). In contrast, nuclear ORAI2 expression showed no significant association with OS or DFS (Fig. S5G,H). Cox multivariate analysis further confirmed that cytoplasmic ORAI2 expression, along with age, pathological grade, and N stage, could serve as an independent prognostic factor (Table S11), whereas nuclear ORAI2 expression could not (Table S12). Spearman correlation analysis revealed a significant positive correlation between Circ72688 and ORAI2 expression, with correlation coefficients of 0.3081 in the cytoplasm (Fig. 4I) and 0.2796 in the nucleus (Fig. 4J). Collectively, these findings indicate that both Circ72688 and ORAI2 are upregulated in breast cancer tissues and positively correlated, and that cytoplasmic ORAI2 expression serves as an independent prognostic factor.

### 3.5 Immune Infiltration, Drug Sensitivity, and Functional Pathway Analysis in High- and Low-Risk Groups

**Figure 5:**
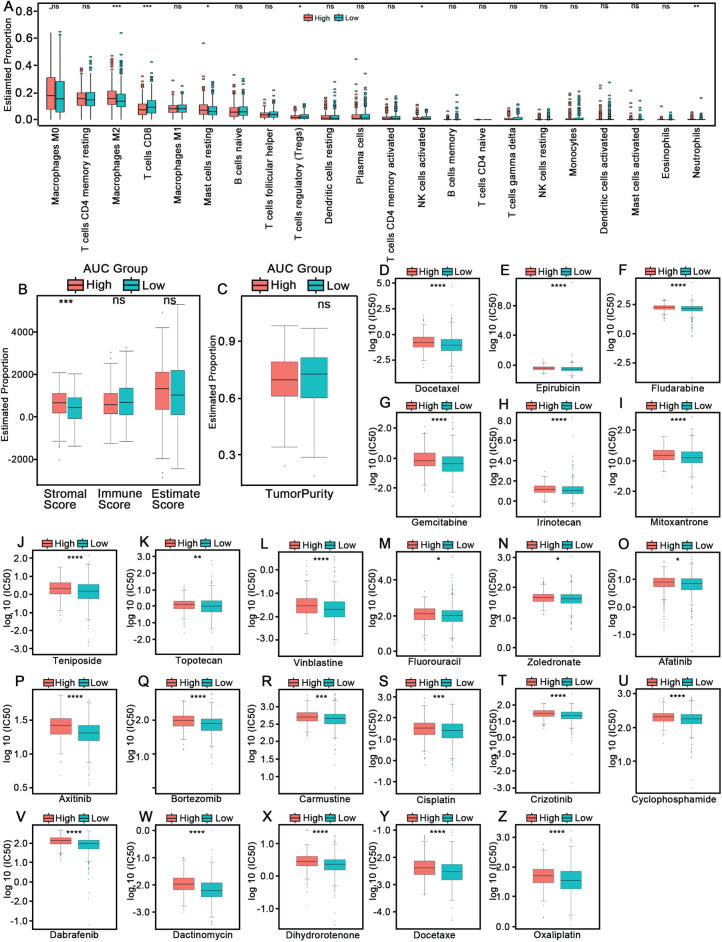
Immune landscape and drug sensitivity differences between high- and low-risk groups. (**A**) Comparison of immune cell infiltration profiles in high- and low-risk groups estimated by the CIBERSORT algorithm. ns: not significant.*Statistical significance: *p < 0.05, **p < 0.01, ***p < 0.001 (**B**) Stromal score, immune score, and ESTIMATE score between the two risk groups.ns: not significant.*Statistical significance: ***p < 0.001 (**C**) Tumor purity comparison between high- and low-risk groups.ns: not significant. (**D**–**Z**) Predicted drug sensitivity (IC50 values) of common chemotherapeutic and targeted agents in the high- and low-risk groups, including Docetaxel (**D**,**Y**), Epirubicin (**E**), Fludarabine (**F**), Gemcitabine (**G**), Irinotecan (**H**), Mitoxantrone (**I**), Teniposide (**J**), Topotecan (**K**), Vinblastine (**L**), 5-Fluorouracil (**M**), Zoledronate (**N**), Afatinib (**O**), Axitinib (**P**), Bortezomib (**Q**), Carmustine (**R**), Cisplatin (**S**), Crizotinib (**T**), Cyclophosphamide (**U**), Dabrafenib (**V**), Dactinomycin (**W**), Dihydrorotenone (**X**), and Oxaliplatin (**Z**). *Statistical significance: *p < 0.05, **p < 0.01, ***p < 0.001, ****p < 0.0001. AUC: Area Under the Curve.

To explore the biological differences between high- and low-risk groups derived from the AUC-based prognostic model, we performed immune cell infiltration analysis using both CIBERSORT and xCell algorithms. The high-risk group exhibited significantly higher proportions of M2 macrophages, resting mast cells, and neutrophils, whereas CD8^+^ T cells, regulatory T cells (Tregs), and activated NK cells were more enriched in the low-risk group (Fig. 5A). These findings suggest that the high-risk group may be characterized by an immunosuppressive tumor microenvironment. xCell analysis further revealed that the high-risk group displayed a significantly higher stromal score, whereas immune score and tumor purity did not differ significantly between the two groups (Fig. 5B,C). To assess the potential therapeutic implications of the risk model, we performed drug sensitivity prediction using the oncoPredict algorithm. The high-risk group exhibited significantly higher predicted IC50 values, indicating poorer sensitivity, to a broad range of commonly used chemotherapeutic and targeted agents, including Docetaxel (Fig. 5D), Epirubicin (Fig. 5E), Fludarabine (Fig. 5F), Gemcitabine (Fig. 5G), Irinotecan (Fig. 5H), Mitoxantrone (Fig. 5I), Teniposide (Fig. 5J), Topotecan (Fig. 5K), Vinblastine (Fig. 5L), 5-Fluorouracil (Fig. 5M), Zoledronate (Fig. 5N), Afatinib (Fig. 5O), Axitinib (Fig. 5P), Bortezomib (Fig. 5Q), Carmustine (Fig. 5R), Cisplatin (Fig. 5S), Crizotinib (Fig. 5T), Cyclophosphamide (Fig. 5U), Dabrafenib (Fig. 5V), Dactinomycin (Fig. 5W), Dihydrorotenone (Fig. 5X), and Oxaliplatin (Fig. 5Z). These results suggest that patients in the high-risk group may have reduced response to multiple therapeutic agents. To further elucidate the underlying biological mechanisms, Gene Set Variation Analysis (GSVA) was performed to assess pathway enrichment. Significant differences in functional pathway enrichment were observed between the high- and low-risk groups across multiple gene set categories, including GOBP (Fig. S6A,B), GOCC (Fig. S6C,D), GOMF (Fig. S6E,F), Hallmark pathways (Fig. S6G,H), and KEGG signaling pathways (Fig. S6I,J).

## 4 Discussion

In this study, we identified Circ72688, a circRNA derived from the ADAMTS6 gene, from circRNA profiling of breast cancer cells and demonstrated that Circ72688 promotes breast cancer progression both *in vitro* and *in vivo*. Mechanistically, Circ72688 partially regulates ORAI2 expression by sponging hsa-miR-654-5p. Both Circ72688 and ORAI2 were highly expressed in breast cancer specimens. Further analyses showed that cytoplasmic ORAI2 expression could predict the prognosis of breast cancer patients, and Circ72688 expression was positively correlated with ORAI2 expression. Using LASSO regression based on Circ72688-related ncRNAs and mRNAs, we constructed a risk model that stratified patients into high- and low-risk groups and found that Circ72688-associated signatures were linked to immune cell infiltration patterns and drug sensitivity in breast cancer.

Circ72688 has previously been reported as a tumor-promoting circRNA in several malignancies. In glioblastoma, under hypoxic conditions, AP-1 and TDP43 promote the biogenesis of Circ72688 from ADAMTS6, thereby enhancing tumor cell growth through the ANXA2/NF-κB signaling pathway [26]. In ESCC, Circ72688 derived from ADAMTS6 activates the Hippo pathway by upregulating AGR2, promoting proliferation and metastasis [24]. Similarly, Circ72688 overexpression has been detected in ICC tumor tissues and plasma exosomes, where it contributes to T-cell exhaustion and neutrophil extracellular trap formation [25]. These studies collectively support a tumor-enhancing and potentially druggable role for Circ72688 across multiple cancer types. Our findings extend this paradigm to breast cancer by demonstrating that Circ72688 selectively binds hsa-miR-654-5p—a miRNA not previously implicated in ADAMTS6-derived circRNA biology—and thereby upregulates ORAI2. ORAI2 is a key calcium channel involved in regulating tumor cell motility. Unlike earlier work focusing predominantly on proliferation and survival, our data indicate that Circ72688 primarily modulates the migratory and invasive behavior of breast cancer cells, at least in part via the miR-654-5p/ORAI2 axis.

In this study, we initially evaluated Circ72688 expression in a breast cancer tissue microarray. However, due to the relatively limited sample size, we were unable to establish a definitive association between Circ72688 expression and clinicopathological parameters or patient prognosis. Increasing the sample size and incorporating multicenter cohorts would help clarify whether Circ72688 can serve as an independent prognostic marker. In addition, integrating Circ72688 expression with established clinical prognostic indicators—such as TNM stage, hormone receptor status, and HER2 status—may facilitate the development of a more comprehensive prognostic model for breast cancer.

Mechanistically, Circ72688 acts as a sponge for hsa-miR-654-5p, which is significantly downregulated in breast cancer. Functional rescue experiments showed that upregulation of hsa-miR-654-5p partially reversed the enhanced migration and invasion induced by Circ72688 overexpression, while inhibition of hsa-miR-654-5p attenuated the suppressive effects of Circ72688 knockdown, supporting a functional Circ72688–miR-654-5p axis. Previously, hsa-miR-654-5p has been reported to inhibit epithelial–mesenchymal transition (EMT) and decrease migratory capacity in lung cancer cells, whereas in oral squamous cell carcinoma it was shown to target GRAP and promote proliferation, metastasis, and chemoresistance via the Ras/MAPK signaling pathway [39–43]. In our study, hsa-miR-654-5p bound to the 3^′^UTR of ORAI2 mRNA, thereby modulating ORAI2 transcription in breast cancer cells. Silencing Circ72688 decreased ORAI2 expression, whereas inhibiting hsa-miR-654-5p partially reversed this effect. Consistent with these *in vitro* results, a positive correlation between Circ72688 and ORAI2 expression was observed in breast cancer specimens.

ORAI2, a calcium channel protein, plays a central role in calcium influx in breast cancer cells. Previous studies have reported that ORAI2 cooperates with SPCA2 to trigger store-independent calcium entry, thereby promoting the proliferation and survival of breast cancer cells [44,45]. Knockdown of ORAI2 induces G0–G1 cell cycle arrest and reduces anti-apoptotic capacity following cisplatin treatment, suggesting a possible role in drug resistance [46]. In line with these reports, our study found that ORAI2 is more highly expressed in breast cancer tissues than in adjacent normal tissues, and that increased cytoplasmic ORAI2 expression is associated with poor prognosis in breast cancer patients.

Using LASSO regression analysis of TCGA data, we developed a risk score model based on Circ72688-related miRNAs and mRNAs. Patients in the high-risk group had worse survival outcomes and displayed higher levels of M2 macrophage infiltration, whereas those in the low-risk group had better survival and showed increased infiltration of CD8^+^ T cells. Previous studies have reported that enrichment of M2 macrophages is indicative of an immunosuppressive tumor microenvironment, while a higher proportion of CD8^+^ T cells is associated with a favorable prognosis [47–51]. Consistently, our GSVA results indicated that multiple immune- and stromal-related pathways differed between risk groups. Furthermore, the high-risk group exhibited reduced predicted sensitivity to a variety of chemotherapeutic agents. Although a direct link between Circ72688 and drug sensitivity has not been previously reported, members of the ADAMTS family have been implicated in modulating therapeutic response. For example, ADAMTS12 promotes chemoresistance in gastric cancer, and ADAMTS6 has been shown to interact with AGR2 in drug-resistant non-small-cell lung cancer [52,53]. In ovarian cancer, mutations in ADAMTS family members have been associated with chemotherapy response [54], and ADAMTS6 overexpression alters cisplatin sensitivity [55]. ADAMTS6 overexpression has also been observed in drug-resistant MCF-7 cells [56]. In our study, Circ72688 is generated from ADAMTS6, but we did not directly demonstrate that Circ72688 alone alters drug sensitivity. Nevertheless, the LASSO-based high-risk group exhibited poorer predicted drug response than the low-risk group, suggesting a potential link between Circ72688-associated networks and chemoresistance. Whether Circ72688 exerts an independent effect on drug resistance requires further investigation.

### 4.1 Future Work

Future studies will aim to clarify the upstream regulatory mechanisms governing Circ72688 biogenesis, including transcriptional and post-transcriptional factors that modulate ADAMTS6 splicing patterns. It will also be important to determine whether Circ72688 participates in additional signaling pathways beyond the miR-654-5p/ORAI2 axis, particularly those related to EMT, metabolic reprogramming, and DNA damage response. Moreover, given the context-dependent roles observed for ORAI2 and miR-654-5p—where their experimentally validated cellular functions do not fully align with their prognostic associations in TCGA cohorts—future work will integrate multi-omics profiling and longitudinal clinical datasets to dissect their stage-specific and microenvironment-dependent functions in breast cancer progression. The use of immune-competent or patient-derived xenograft and organoid models will also be critical to explore how Circ72688 influences the tumor immune microenvironment and therapeutic response in more clinically relevant settings.

### 4.2 Limitations

This study has several limitations. First, although Circ72688 was functionally validated using *in vitro* assays and *in vivo* metastasis models, its clinical significance was primarily assessed using retrospective TCGA-based datasets, which may be affected by incomplete follow-up, missing treatment information, and potential informative censoring, thereby limiting the robustness of gene–survival associations [57,58]. Consequently, discrepancies were observed in the prognostic analyses of miR-654-5p and ORAI2. Specifically, miR-654-5p was expressed at a lower level in tumor tissues than in normal controls, yet higher miR-654-5p expression was associated with worse prognosis; conversely, ORAI2 was highly expressed in tumor tissues, but higher ORAI2 expression was unexpectedly correlated with better survival outcomes in some TCGA-based analyses. Second, although our experimental work focused on the metastasis-related functions of Circ72688, other potential biological roles—such as effects on proliferation, immune modulation, and therapeutic response—as well as additional downstream targets and signaling pathways were not comprehensively explored. Third, immune- and therapy-related effects inferred from bioinformatic analyses (e.g., immune infiltration, drug sensitivity prediction, and pathway enrichment) were not experimentally validated *in vivo* and remain to be confirmed in clinically relevant models [59,60].

Collectively, these limitations indicate that further validation in well-annotated clinical cohorts, together with multi-dimensional functional studies, is required to fully delineate the clinical and biological importance of the Circ72688–miR-654-5p–ORAI2 axis in breast cancer.

## 5 Conclusion

In conclusion, this study suggested that Circ72688 acted as an oncogene in breast cancer. Overexpressed Circ72688 sponged hsa-miR-654-5p and thereby partially regulated ORAI2. Circ72688 might serve as a potential therapeutic target for breast cancer.

## Supplementary Materials



## Data Availability

The circRNA sequencing data generated in this study cannot be openly shared at this stage because the research team plans to use them for further studies. The data supporting the findings of this study are available from the corresponding author, Fengtao Ji, upon reasonable request. All publicly analyzed bioinfor-matics datasets (including TCGA and GEO cohorts) are openly accessible, and the corresponding database URLs and accession numbers have been clearly provided in the Methods section.

## Supplementary Materials

The supplementary material is available online at https://www.techscience.com/doi/10.32604/or.2026.073081/s1.
