# Supplementary material for: Circ72688 Drives Breast Cancer Invasion and Metastasis via the miR-654-5p/ORAI2 Axis
Source: Oncol Res. 2026 Apr 22;34(5):33. doi: 10.32604/or.2026.073081 (PMC13126400; doi:10.32604/or.2026.073081)
Supplement: Supplementary file 1 [file OncolRes-34-73081-s001.doc]

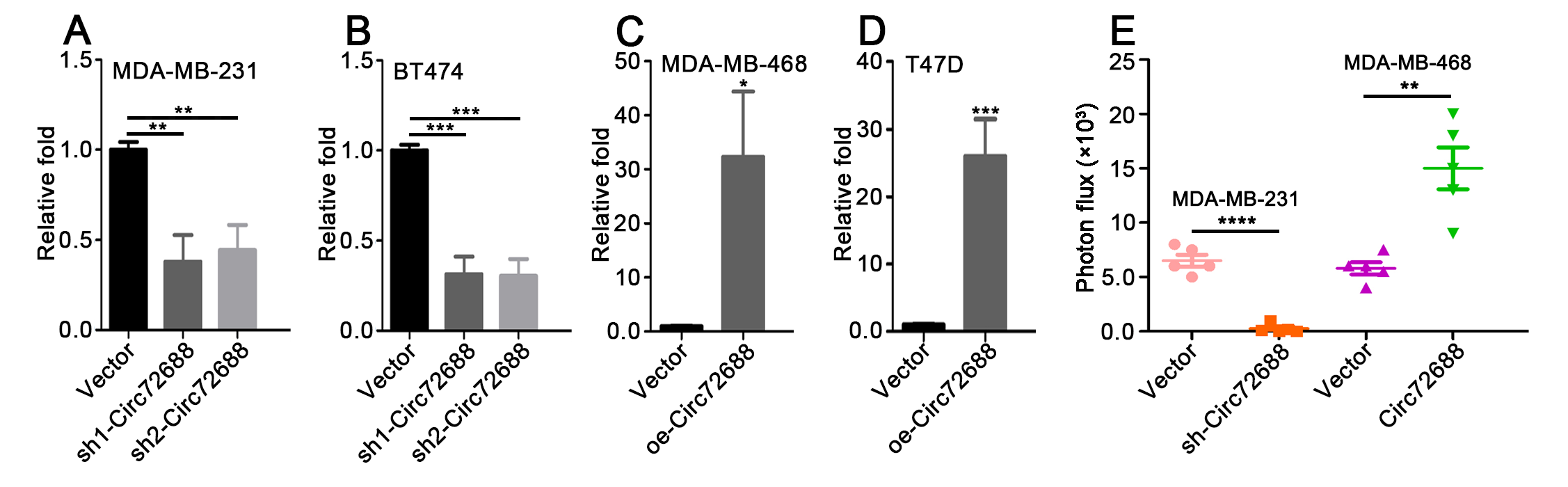


**Figure S1. Regulation of Circ72688 expression in breast cancer cell line.** (A-B) qRT-PCR to detect the efficiency of lentiviral mediated Circ72688 silencing. (C-D) qRT-PCR to detect the efficiency of lentiviral mediated Circ72688 silencing. (E) Quantification of bioluminescent signal intensity from in vivo imaging in different experimental groups. Statistical significance: *p < 0.05, **p < 0.01, ***p < 0.001, ****p < 0.0001. qRT-PCR: Quantitative Reverse Transcription Polymerase Chain Reaction; sh: short hairpin RNA.


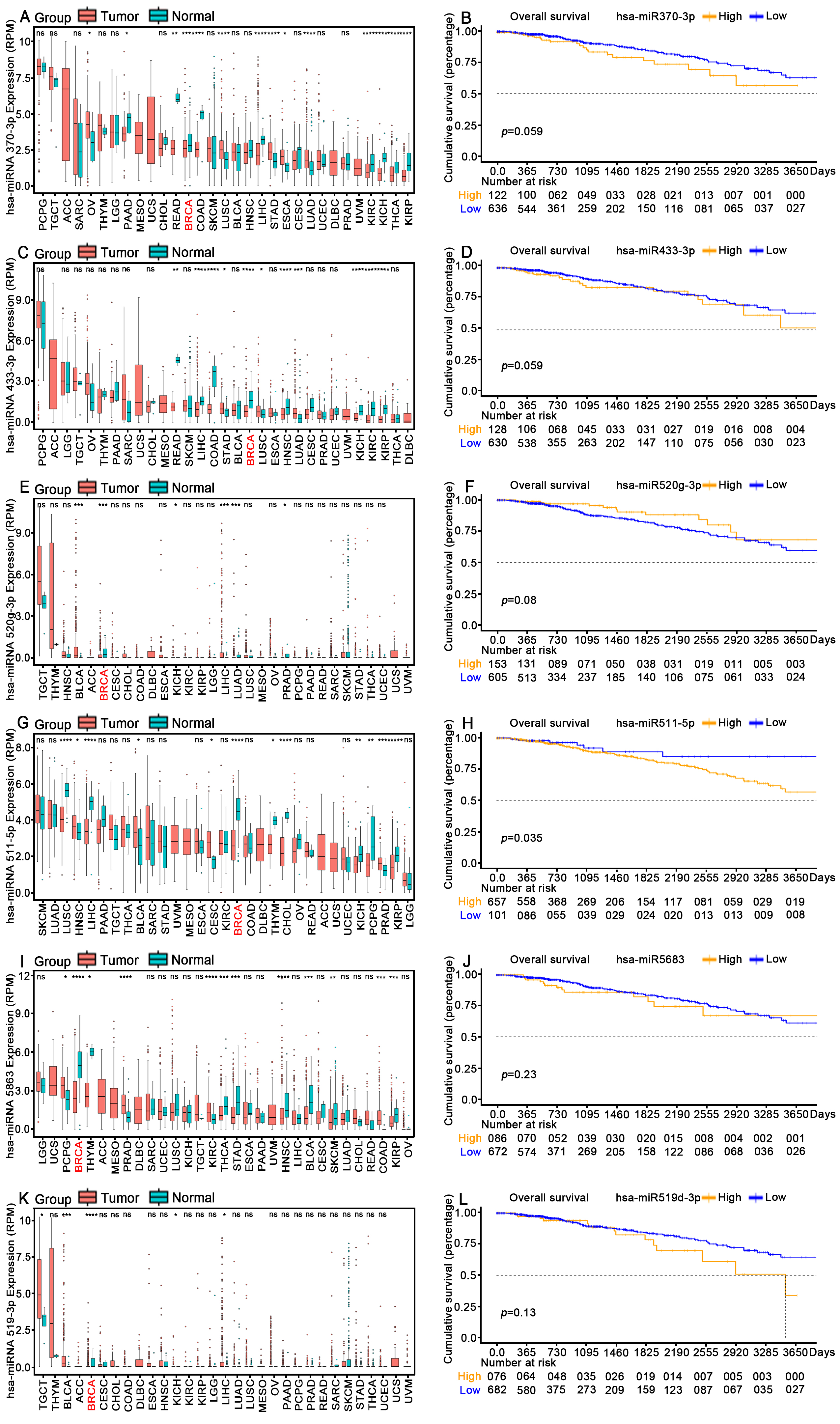


**Figure S2. Pan-cancer analysis of miRNAs and prognosis in breast cancer. (**A-B) Pan-cancer analysis of hsa-miR-370-3p and prognosis in breast cancer. (C-D) Pan-cancer analysis of hsa-miR-433-3p and prognosis in breast cancer. (E-F) Pan-cancer analysis of hsa-miR-520g-3p and prognosis in breast cancer. (G-H) Pan-cancer analysis of hsa-miR-511-5p and prognosis in breast cancer. (I-J) Pan-cancer analysis of hsa-miR-5683 and prognosis in breast cancer. (K-L) Pan-cancer analysis of hsa-miR-519d-3p and prognosis in breast cancer. Statistical significance: *p < 0.05, **p < 0.01, ***p < 0.001, ****p < 0.0001. ns: no significance


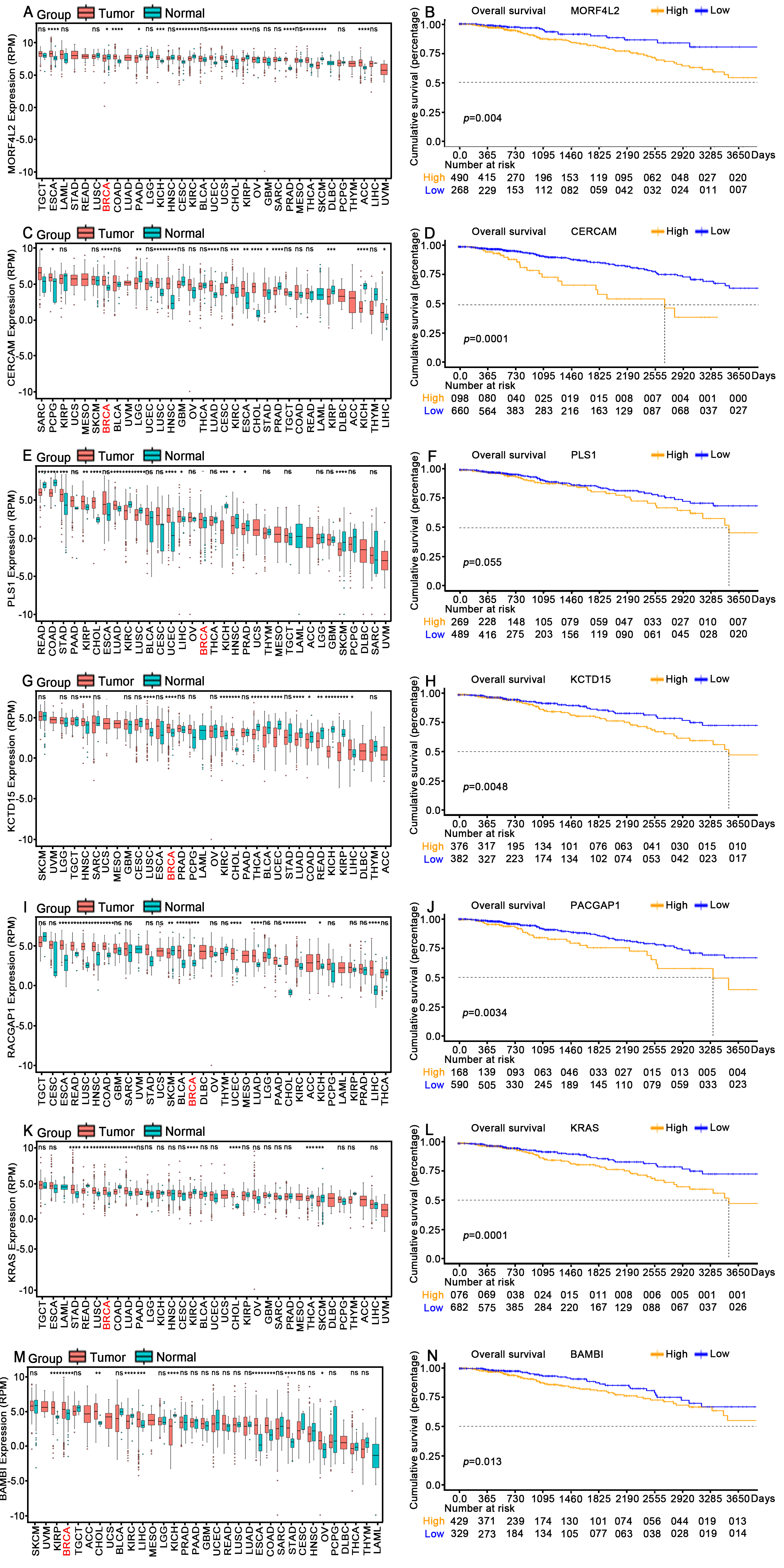


**Figure S3. Pan-cancer analysis of mRNAs and prognosis in breast cancer. (**A-B) Pan-cancer analysis of MORF4L2 and prognosis in breast cancer. (C-D) Pan-cancer analysis of CERCAM and prognosis in breast cancer. (E-F) Pan-cancer analysis of PLS1 and prognosis in breast cancer. (G-H) Pan-cancer analysis of KCTD15 and prognosis in breast cancer. (I-J) Pan-cancer analysis of RACGAP1 and prognosis in breast cancer. (K-L) Pan-cancer analysis of KRAS and prognosis in breast cancer. (M-N) Pan-cancer analysis of BAMBI and prognosis in breast cancer. *p < 0.05, **p < 0.01, ***p < 0.001, ****p < 0.0001. ns: no significance


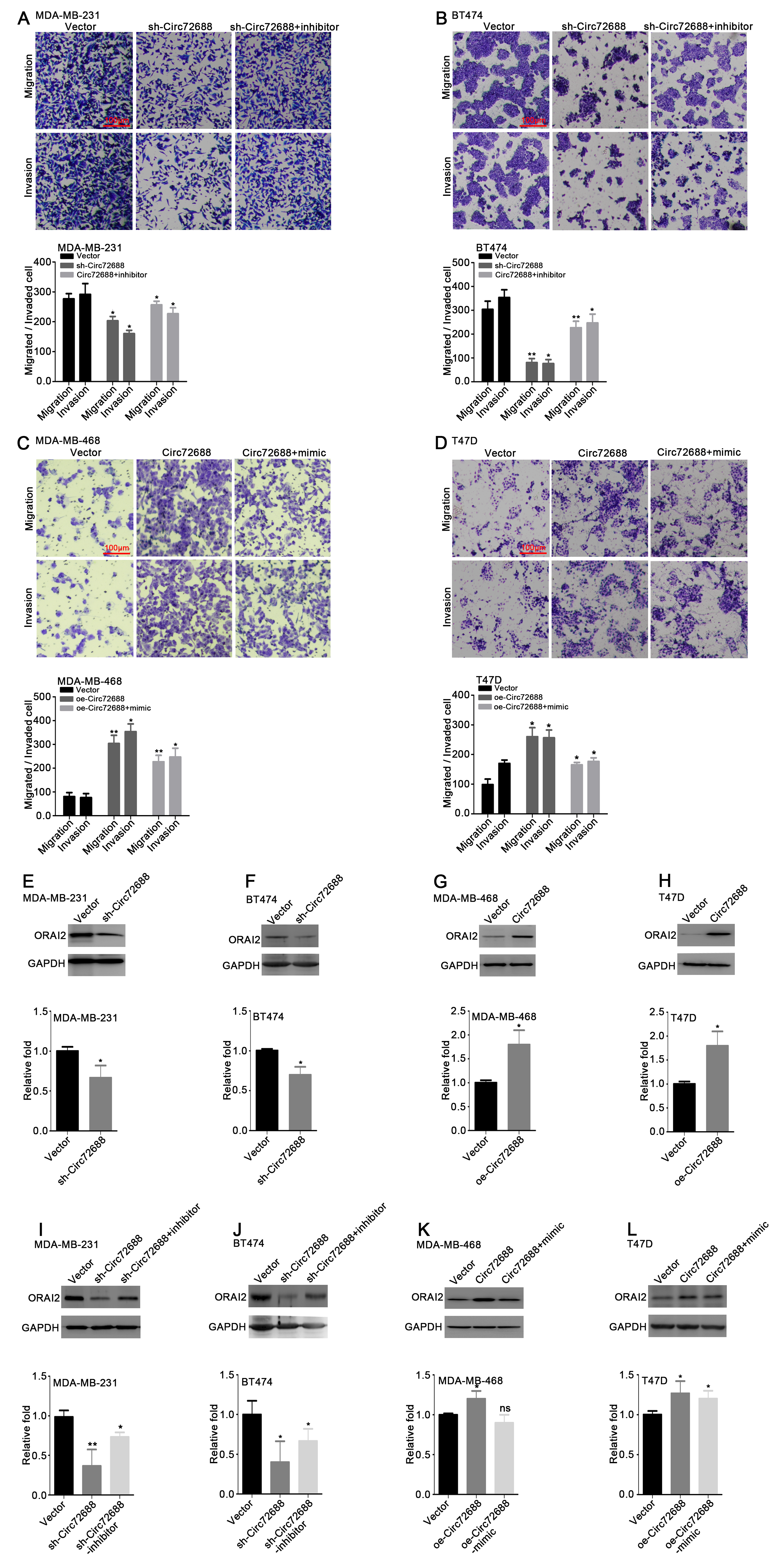


**Figure S4. Circ72688 Regulates ORAI2 via hsa-miR654-5p to Promote Breast Cancer Cell Migration and Invasion** (A) Transwell assay to detect the migration and invasion abilities of MDA-MB-231 cells with Circ72688 silencing and hsa-miR654-5p inhibitor co-transfection. (B) Transwell assay to detect the migration and invasion abilities of BT474 cells with Circ72688 silencing and hsa-miR654-5p inhibitor co-transfection. (C) Transwell assay to detect the migration and invasion abilities of MDA-MB-468 cells with Circ72688 overexpression and hsa-miR654-5p mimic co-transfection. (D) Transwell assay to detect the migration and invasion abilities of T47D cells with Circ72688 overexpression and hsa-miR654-5p mimic co-transfection. (E) Western blot to detect ORAI2 expression in MDA-MB-231 cells after Circ72688 silencing. (F) Western blot to detect ORAI2 expression in BT474 cells after Circ72688 silencing. (G) Western blot to detect ORAI2 expression in MDA-MB-468 cells after Circ72688 overexpression. (H) Western blot to detect ORAI2 expression in T47D cells after Circ72688 overexpression. (I) Western blot to detect ORAI2 expression in MDA-MB-231 cells with Circ72688 silencing and hsa-miR654-5p inhibitor co-transfection. (J) Western blot to detect ORAI2 expression in BT474 cells with Circ72688 silencing and hsa-miR654-5p inhibitor co-transfection. (K) Western blot to detect ORAI2 expression in MDA-MB-468 cells with Circ72688 overexpression and hsa-miR654-5p mimic co-transfection. (L) Western blot to detect ORAI2 expression in T47D cells with Circ72688 overexpression and hsa-miR654-5p mimic co-transfection. *p < 0.05, **p < 0.01. ns: no significance; sh: short hairpin RNA.


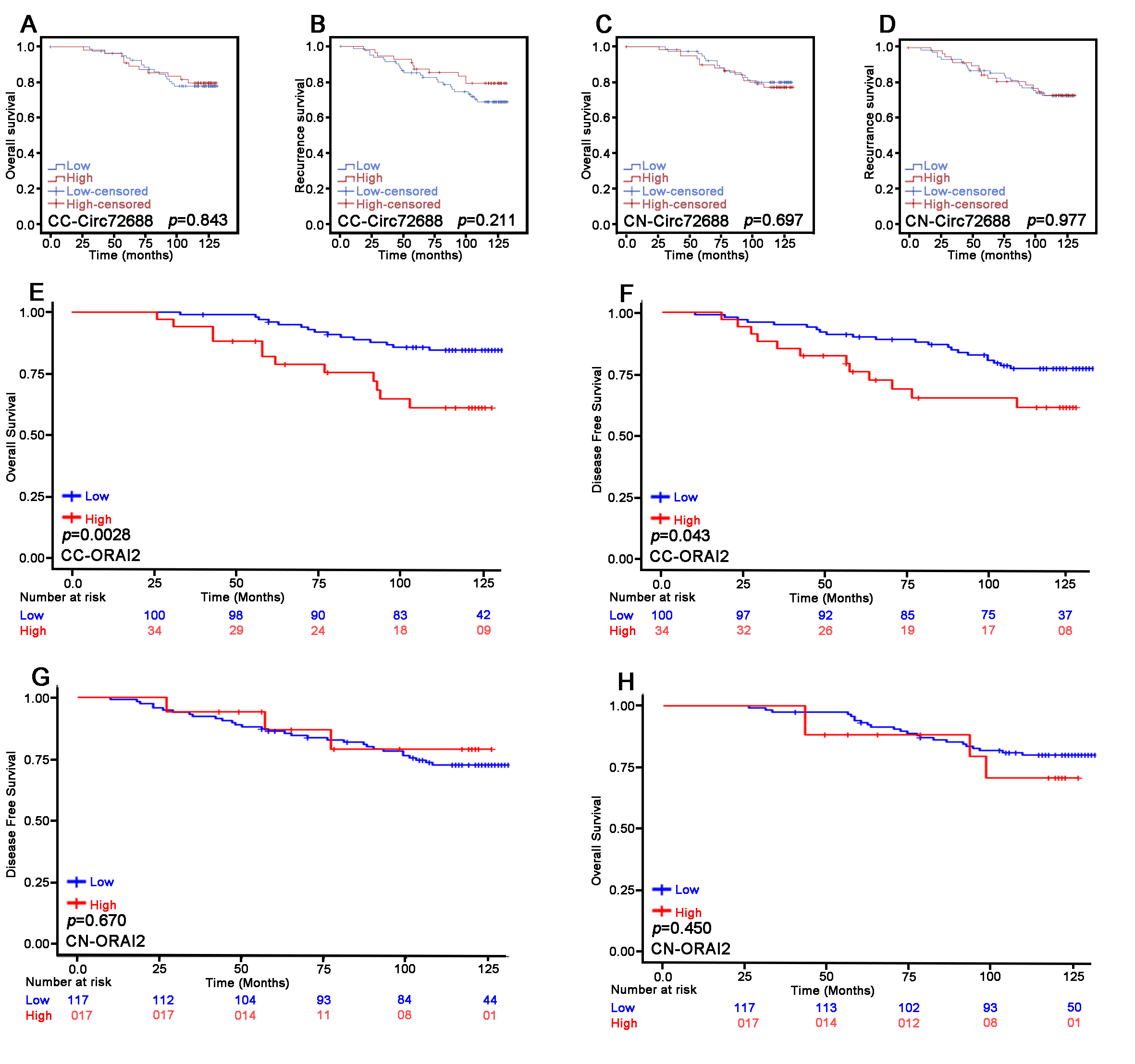


**Figure S5. Relationship between Circ72688 and ORAI2 expression levels and prognosis.** (A) KM survival analysis of cytoplasmic Circ72688 expression and OS. (B) KM survival analysis of cytoplasmic Circ72688 expression and RS. (C) KM survival analysis of nuclear Circ72688 expression and OS. (D) KM survival analysis of nuclear Circ72688 expression and RS. (E) KM survival analysis of cytoplasmic ORAI2 expression and OS. (F) KM survival analysis of cytoplasmic ORAI2 expression and DFS. (G) KM survival analysis of nuclear ORAI2 expression and OS. (H) KM survival analysis of nuclear ORAI2 expression and DFS. KM: Kaplan-Meier

**Table S1** Differential expression of Circ72688 in the cytoplasm of cancer and adjacent tissues

|  | n | Circ72688 expression | |
| --- | --- | --- | --- |
|  |  | High | Low |
| Cancer tissues | 64 | 28 | 36 |
| Adjacent tissues | 64 | 3 | 61 |

**Table S2** Differential expression of Circ72688 in the nucleus of cancer and adjacent tissues

|  | n | Circ72688 expression | |
| --- | --- | --- | --- |
|  |  | High | Low |
| Cancer tissues | 64 | 28 | 36 |
| Adjacent tissues | 64 | 6 | 58 |

**Table S3** Correlation Analysis of Circ72688 with Clinical Parameters in Cancer Patients: Chi-square Test

Correlation between Circ72688 expression in the cytoplasm and clinicopathological characteristics

|  | Variables | Circ72688 expression | | total | χ2 | *p* value |
| --- | --- | --- | --- | --- | --- | --- |
|  |  | low | high |  |  |  |
| Age(year) |  |  |  |  | 1.024 | 0.311 |
|  | ≤55 | 42 | 33 | 75 |  |  |
|  | >55 | 40 | 22 | 62 |  |  |
| Grade stage |  |  |  |  | 0.108 | 0.743 |
|  | II | 66 | 43 | 109 |  |  |
|  | III | 16 | 12 | 28 |  |  |
| TNM stage |  |  |  |  | 0.122 | 0.727 |
|  | Ι | 20 | 12 | 32 |  |  |
|  | II/III | 62 | 43 | 105 |  |  |
| T stage |  |  |  |  | 0.547 | 0.460 |
|  | T1 | 35 | 20 | 55 |  |  |
|  | T2/T3 | 47 | 35 | 82 |  |  |
| N stage |  |  |  |  | 0.897 | 0.344 |
|  | N0 | 47 | 27 | 74 |  |  |
|  | N1/N2/N3 | 35 | 28 | 63 |  |  |

Footnote: TNM: Tumor-Node-Metastasis; T: Tumor; N: Node; cADAMTS6: circular ADAMTS6; *statistically significant (p <0.05).

**Table S4** Correlation between Circ72688 expression in the nuclear and clinicopathological characteristics

|  | variables | Circ72688 expression | | total | χ2 | *p* value |
| --- | --- | --- | --- | --- | --- | --- |
|  |  | low | high |  |  |  |
| Age(year) |  |  |  |  | 0.086 | 0.770 |
|  | ≤55 | 43 | 32 | 75 |  |  |
|  | >55 | 34 | 28 | 62 |  |  |
| Grade stage |  |  |  |  | 0.550 | 0.458 |
|  | II | 63 | 46 | 109 |  |  |
|  | III | 14 | 14 | 28 |  |  |
| TNM stage |  |  |  |  | 0.171 | 0.680 |
|  | Ι | 19 | 13 | 32 |  |  |
|  | II/III | 58 | 47 | 105 |  |  |
| T stage |  |  |  |  | 0.001 | 0.975 |
|  | T1 | 31 | 24 | 55 |  |  |
|  | T2/T3 | 46 | 36 | 82 |  |  |
| N stage |  |  |  |  | 2.320 | 0.128 |
|  | N0 | 46 | 28 | 72 |  |  |
|  | N1/N2/N3 | 31 | 32 | 63 |  |  |

Footnote: TNM: Tumor-Node-Metastasis; T: Tumor; N: Node; *statistically significant (p <0.05).

**Table S5** Univariate and multivariate analyses of the factors correlated with Overall survival of Breast carcinoma patients

Variables in the Equation (Circ72688 in the cytoplasm)

| variables | Univariate analysis | | |  | Multivariate analysis | | |
| --- | --- | --- | --- | --- | --- | --- | --- |
|  | HR | 95%CI | *p* value |  | HR | 95%CI | *p* value |
| expression | 0.926 | 0.434-1.978 | 0.843 |  |  |  |  |
| Age | 2.420 | 1.142-5.129 | 0.021 |  | 2.926 | 1.367-6.262 | 0.006 |
| Grade stage | 2.617 | 1.235-5.543 | 0.012 |  | 2.550 | 1.196-5.436 | 0.015 |
| TNM stage | 2.954 | 0.894-9.764 | 0.076 |  |  |  |  |
| T stage | 1.312 | 0.610-2.822 | 0.487 |  |  |  |  |
| N stage | 2.789 | 1.270-6.128 | 0.011 |  | 2.906 | 1.312-6.439 | 0.009 |

Footnote: TNM: Tumor-Node-Metastasis; T: Tumor; N: Node; HR: Hazard Ratio; CI: Confidence Interval; *statistically significant (p <0.05).

**Table S6** Univariate and multivariate analyses of the factors correlated with Overall survival of Breast carcinoma patients

Variables in the Equation (Circ72688 in the nucleus)

| variables | Univariate analysis | | |  | Multivariate analysis | | |
| --- | --- | --- | --- | --- | --- | --- | --- |
|  | HR | 95%CI | *p* value |  | HR | 95%CI | *p* value |
| expression | 1.159 | 0.551-2.436 | 0.697 |  |  |  |  |
| Age | 2.420 | 1.142-5.129 | 0.021 |  | 2.926 | 1.367-6.262 | 0.006 |
| Grade stage | 2.617 | 1.235-5.543 | 0.012 |  | 2.550 | 1.196-5.436 | 0.015 |
| TNM stage | 2.954 | 0.894-9.764 | 0.076 |  |  |  |  |
| T stage | 1.312 | 0.610-2.822 | 0.487 |  |  |  |  |
| N stage | 2.789 | 1.270-6.128 | 0.011 |  | 2.906 | 1.312-6.439 | 0.009 |

Footnote: TNM: Tumor-Node-Metastasis; T: Tumor; N: Node; HR: Hazard Ratio; CI: Confidence Interval; *statistically significant (p <0.05).

**Table S7** Differential expression of ORAI2 in cytoplasm of cancer and adjacent tissues

|  | n | ORAI2 expression | |
| --- | --- | --- | --- |
|  |  | High | Low |
| Cancer tissues | 64 | 19 | 45 |
| Adjacent tissues | 64 | 6 | 58 |

Footnote: ORAI2: ORAI calcium release-activated calcium modulator 2

**Table S8** Differential expression of ORAI2 in nucleus of cancer and adjacent tissues

|  | n | ORAI2 expression | |
| --- | --- | --- | --- |
|  |  | High | Low |
| Cancer tissues | 64 | 8 | 56 |
| Adjacent tissues | 64 | 0 | 64 |

Footnote: ORAI2: ORAI calcium release-activated calcium modulator 2

**Table S9** Correlation between ORAI2 expression in the cytoplasm of tumor tissue and clinicopathological characteristics

|  | variables | ORAI2 expression | | total | χ2 | *p* value |
| --- | --- | --- | --- | --- | --- | --- |
|  |  | low | high |  |  |  |
| Age(year) |  |  |  |  | 0.103 | 0.748 |
|  | ≤55 | 54 | 20 | 74 |  |  |
|  | >55 | 46 | 15 | 61 |  |  |
| Grade stage |  |  |  |  | 2.170 | 0.141 |
|  | II | 77 | 31 | 108 |  |  |
|  | III | 23 | 4 | 27 |  |  |
| TNM stage |  |  |  |  | 0.000 | 0.986 |
|  | Ι | 23 | 8 | 31 |  |  |
|  | II/III | 77 | 27 | 104 |  |  |
| T stage |  |  |  |  | 0.161 | 0.688 |
|  | T1 | 39 | 15 | 54 |  |  |
|  | T2/T3 | 61 | 20 | 81 |  |  |
| N stage |  |  |  |  | 0.069 | 0.793 |
|  | N0 | 54 | 18 | 72 |  |  |
|  | N1/N2/N3 | 46 | 17 | 63 |  |  |

Footnote: TNM: Tumor-Node-Metastasis; T: Tumor; N: Node; HR: Hazard Ratio; CI: Confidence Interval; ORAI2: ORAI calcium release-activated calcium modulator 2; *statistically significant (p <0.05).

**Table S10** Correlation between ORAI2 expression in the nucleus and clinicopathological characteristics

|  | variables | ORAI2 expression | | total | χ2 | *p* value |
| --- | --- | --- | --- | --- | --- | --- |
|  |  | low | high |  |  |  |
| Age(year) |  |  |  |  | 0.126 | 0.722 |
|  | ≤55 | 64 | 10 | 74 |  |  |
|  | >55 | 54 | 7 | 61 |  |  |
| Grade stage |  |  |  |  | 0.067 | 0.795 |
|  | II | 94 | 14 | 108 |  |  |
|  | III | 24 | 3 | 27 |  |  |
| TNM stage |  |  |  |  | 0.004 | 0.953 |
|  | Ι | 27 | 4 | 31 |  |  |
|  | II/III | 91 | 13 | 104 |  |  |
| T stage |  |  |  |  | 0.972 | 0.324 |
|  | T1 | 44 | 10 | 54 |  |  |
|  | T2/T3 | 74 | 7 | 81 |  |  |
| N stage |  |  |  |  | 1.155 | 0.283 |
|  | N0 | 65 | 7 | 72 |  |  |
|  | N1/N2/N3 | 53 | 10 | 63 |  |  |

Footnote: TNM: Tumor-Node-Metastasis; T: Tumor; N: Node; HR: Hazard Ratio; CI: Confidence Interval; ORAI2: ORAI calcium release-activated calcium modulator 2; *statistically significant (p <0.05).

**Table S11** Univariate and multivariate analyses of the factors correlated with Overall survival of Breast carcinoma patients

Variables in the Equation (ORAI2 in the cytoplasm)

| variables | Univariate analysis | | |  | Multivariate analysis | | |
| --- | --- | --- | --- | --- | --- | --- | --- |
|  | HR | 95%CI | *p* value |  | HR | 95%CI | *p* value |
| expression | 3.016 | 1.409-6.456 | 0.004 |  | 0.307 | 0.115-0.819 | 0.000 |
| Age | 2.420 | 1.142-5.129 | 0.021 |  | 2.683 | 1.214-5.931 | 0.004 |
| Grade stage | 2.617 | 1.235-5.543 | 0.012 |  | 2.125 | 0.965-4.682 | 0.004 |
| TNM stage | 2.954 | 0.894-9.764 | 0.076 |  |  |  |  |
| T stage | 1.312 | 0.610-2.822 | 0.487 |  |  |  |  |
| N stage | 2.789 | 1.270-6.128 | 0.011 |  | 2.816 | 1.216-6.520 | 0.013 |

Footnote: TNM: Tumor-Node-Metastasis; T: Tumor; N: Node; HR: Hazard Ratio; CI: Confidence Interval; ORAI2: ORAI calcium release-activated calcium modulator 2; *statistically significant (p <0.05).

**Table S12** Univariate and multivariate analyses of the factors correlated with Overall survival of Breast carcinoma patients

Variables in the Equation (ORAI2 in the nucleus)

| variables | Univariate analysis | | |  | Multivariate analysis | | |
| --- | --- | --- | --- | --- | --- | --- | --- |
|  | HR | 95%CI | *p* value |  | HR | 95%CI | *p* value |
| expression | 1.500 | 0.518-4.343 | 0.455 |  |  |  |  |
| Age | 2.420 | 1.142-5.129 | 0.021 |  | 2.926 | 1.367-6.262 | 0.006 |
| Grade stage | 2.617 | 1.235-5.543 | 0.012 |  | 2.550 | 1.196-5.436 | 0.015 |
| TNM stage | 2.954 | 0.894-9.764 | 0.076 |  |  |  |  |
| T stage | 1.312 | 0.610-2.822 | 0.487 |  |  |  |  |
| N stage | 2.789 | 1.270-6.128 | 0.011 |  | 2.906 | 1.312-6.439 | 0.009 |

Footnote: TNM: Tumor-Node-Metastasis; T: Tumor; N: Node; HR: Hazard Ratio; CI: Confidence Interval; ORAI2: ORAI calcium release-activated calcium modulator 2; *statistically significant (p <0.05).


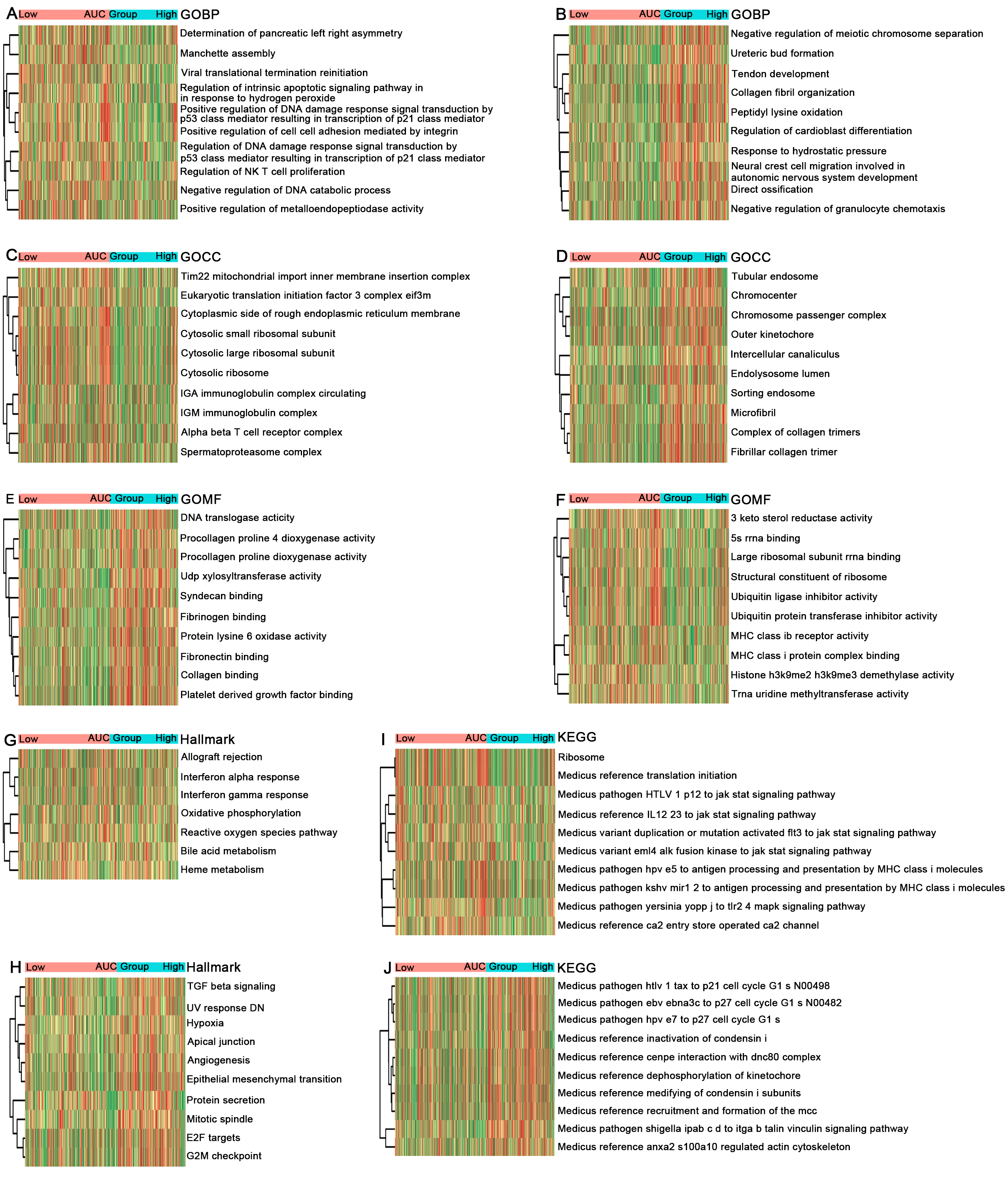


**Figure S6.** **GSVA analysis in the high-risk group and low-risk group. (**A-B) GOBP analysis of differentially expressed genes between the low-risk model group and high-risk model group. (C-D) GOCC analysis of differentially expressed genes between the low-risk model group and high-risk model group. (E-F) GOMF analysis of differentially expressed genes between the low-risk model group and high-risk model group. (G-H) Differentially expressed hallmarks between the low-risk model group and high-risk model group. (I-J) KEGG signaling pathway analysis between the low-risk model group and high-risk model group. GSVA: Gene Set Variation Analysis; GOCC: Gene Ontology Cellular Component; GOMF: Gene Ontology Molecular Function; KEGG: Kyoto Encyclopedia of Genes and Genomes;
